# The Dark Side Is Not Fastidious – Dark Septate Endophytic Fungi of Native and Invasive Plants of Semiarid Sandy Areas

**DOI:** 10.1371/journal.pone.0032570

**Published:** 2012-02-29

**Authors:** Dániel G. Knapp, Alexandra Pintye, Gábor M. Kovács

**Affiliations:** 1 Eötvös Loránd University, Institute of Biology, Department of Plant Anatomy, Budapest, Hungary; 2 Plant Protection Institute, Centre for Agricultural Research, Hungarian Academy of Sciences, Budapest, Hungary; Jyväskylä University, Finland

## Abstract

Dark septate endophytic (DSE) fungi represent a frequent root-colonizing fungal group common in environments with strong abiotic stress, such as (semi)arid ecosystems. This work aimed to study the DSE fungi colonizing the plants of semiarid sandy grasslands with wood steppe patches on the Great Hungarian Plain. As we may assume that fungi colonizing both invasive and native species are generalists, root associated fungi (RAF) were isolated from eight native and three invasive plant species. The nrDNA sequences of the isolates were used for identification. To confirm that the fungi were endophytes an artificial inoculation system was used to test the isolates: we considered a fungus as DSE if it colonized the roots without causing a negative effect on the plant and formed microsclerotia in the roots. According to the analyses of the ITS sequence of nrDNA the 296 isolates clustered into 41 groups. We found that 14 of these 41 groups were DSE, representing approximately 60% of the isolates. The main DSE groups were generalist and showed no specificity to area or season and colonized both native and invasive species, demonstrating that exotic plants are capable of using the root endophytic fungi of the invaded areas. The DSE community of the region shows high similarity to those found in arid grasslands of North America. Taking into account a previous hypothesis about the common root colonizers of those grasslands and our results reported here, we hypothesize that plants of (semi)arid grasslands share common dominant members of the DSE fungal community on a global scale.

## Introduction

Endophytes, which consist of living organisms that colonize plant tissues during some period of their life cycle yet cause no symptoms of tissue damage to their hosts [1], [2], are found in all biomes. Among these endophytes, fungi commonly play important roles in ecosystem functioning [3]. Some fungal endophytic interactions have been widely studied due to the general interest in economically important hosts (e.g., tall fescue) or fungi (e.g., clavicipitaceous fungi) [4]. Although there is an increasing interest in fungal endophytes, our knowledge is biased toward grasses and their above-ground tissues [2].

Dark septate endophytes (DSE) are found worldwide and comprise a group of root-colonizing endophytic fungi that belong to a few orders of the phylum Ascomycota [5]. DSE fungi are septate and generally have melanized hyphae that colonize the cortical cells and intercellular regions of roots and form a densely septated intracellular structure called microsclerotia [5], [6]. Historically, there have been several ambiguities in research on DSE fungi regarding the terms, structures or functions of DSE-plant interactions (see [5]). Although there has been a continual increase in interest in DSE fungi (e.g., [7]–[11]), our knowledge of DSE fungi diversity and their function in ecosystems is limited and not as well understood as that of the common root colonizer mycorrhizal fungi or the previously mentioned grass endophytes.

Recently, Mandyam and Jumponnen [12] reviewed studies on the distribution and frequency of DSE interactions in plant communities of different ecosystems. Although it seems that DSE fungi are present in all major biome types and climate regions, studies on their presence and diversity is sporadic. One of the pioneering and landmark studies of DSE fungi focusing on alpine biomes [13] hypothesized that the abundance and importance of plant-DSE fungi interactions increase with increasing abiotic stress on the environment. This hypothesis was integrated into the scientific canon and was strengthened by the general view that melanin content is related to abiotic stress, especially drought stress [14]. Fungi with melanized cell walls were used in experimental studies showing increased resistance to heat and drought stress (e.g., [15]–[17]). These data and the presence of these fungi in certain habitats resulted in the general view that DSE fungi could play important roles in ecosystems with low water availability. Several studies on root-colonizing fungi of such areas – majority of them represents North American biomes – focused on DSE and other root associated fungi (RAF) (e.g., [18]–[20]).

In the mycorrhizal status studies of plant communities of the Great Hungarian Plain, the majority of the plant species was found to be colonized by DSE fungi [21], [22]. One of the areas studied is a semiarid open sandy grassland with forest steppe patches, and represents a characteristic ecosystem of the plain of the interfluves of the two main rivers of the Carpathian basin, the Danube and Tiscia. From a botanical point of view, this is one of the most studied habitats of the region; in addition to the lengthy vegetation studies [23], [24], one of the three long-term ecological research (LTER) projects in Hungary has been performed here [24]. These grasslands are the westernmost representation of the Eurasian steppe belt [25]. The soil is Danube-origin loose sand with very low organic matter content and almost no clay content. Thus, it has very limited water-holding capacity that results in a harsh environment, especially during the hot summers (Fig. S1). The area is considered semiarid, and the open grasslands have semi-desert characteristics [26]. During the mycorrhizal status studies in this semiarid sandy grassland, 89 plant species were studied; 63 of these were found to be colonized by melanized, septate hyphae, and microsclerotia were detected in 36 species [22]. Due to the frequent colonization, we assumed that DSE fungi play an important role in this ecosystem.

These results prompted us to study the compositional diversity of DSE fungi colonizing the plants of these semiarid sandy areas. We aimed to test the area specificity, the seasonality and whether the DSE fungi were generalist plant colonizers in the area. To test the latter, we sampled invasive plants in addition to the native indigenous hosts. The effects of soil microbiota on invasive plants are generally established [27]–[30]. We may assume that a successful invasive species either do not have a mutualistic partner or can establish functioning interactions not exclusively with specific partners. From a mycocentric standpoint, we assumed that RAF colonizing both the native and invasive plants of the habitat are generalists. The fungal isolates were subjected to molecular taxonomic characterization and used in an artificial synthesis experiment to test whether they could be considered DSE fungi.

## Materials and Methods

### Sampling

The samples were collected from three sites on the Great Hungarian Plain (Hungary); Bugac (N 46°39′ E 19°36′), Fülöpháza (N 46°52′ E 19°24′) and Tatárszentgyörgy (N 47°3′ E 19°24′) (Fig. S1). The main vegetation types are the open grassland at Fülöpháza (*Festucetum vaginatae*) and mixed woody steppe (*Junipero-Populetum*) that are characteristic at each site. The first two areas are protected and belong to the Kiskunság National Park. The three areas are at 100–150 m altitude, the sum of sunny hours/year is 2000–2100, the mean annual precipitation is between 500 and 600 mm/year and the mean annual temperature is 10–12°C [31], [32]. The soils are sandy, rarely mixed with loess, humus contents are less than 1% and the pH is between 7.5 and 8.2.

The roots of eleven plant species were collected in the area, including three invasive species (tree of heaven, *Ailanthus altissima*; common ragweed, *Ambrosia artemisiifolia*; and common milkweed, *Asclepias syriaca*) and eight native species (*Ephedra dystachia*, *Festuca vaginata*, *Fumana procumbens*, *Helianthemum ovatum*, *Juniperus communis*, *Medicago minima*, *Populus alba* and *Stipa borysthenica*). These plant species were chosen because they represent different life forms and they are characteristic components of these plant communities. To test season- and area-specificity, samples were collected from 10 marked *Juniperus communis* trees from each of the three areas in three seasons (spring, summer and fall) of 2008 and 2009.

Samples were taken from healthy-looking plants. The complete root systems of small plant specimens were removed. Otherwise, either the roots were followed from stems and collected or 10×10×10 cm soil cubes were sampled under the plants and roots were identified according to their characteristics. After transporting the roots in soil in plastic bags to the laboratory, the samples were stored at 8°C for no longer than three days.

Between 2005 and 2009, samples were taken three to four times per year from randomly chosen specimens. Invasive species were sampled in the native plant communities or in their close proximities. For example, ragweed generally colonizes disturbed areas and dirt roads but does not invade stable communities. For some species, only a few specimens were sampled (e.g., the strictly protected *E. distachia* and *S. borysthenica*).

### Isolation of root associated fungi

Roots were washed with tap water and cleaned with paint brushes. Three or four segments (1–2 cm long) were cut from different regions of each root sample. The segments were surface-sterilized by washing in 2% sodium hypochlorite (90 s), 70% ethanol (60 s) and sterile distilled water (180 s). Then, the segments were cut into four pieces and placed on MMN media [33] (15 g/L agarose, pH 5.8, 10 µg/ml streptomycin) and kept at 18°C in the dark.

Hyphae growing out of the root segments were isolated to separate plates. Endophytes from the same root sample that showed similar colony morphology were considered identical. The isolates were kept on MMN media in the dark at 18°C, and transferred to new plates every three months.

### Molecular identification of the isolates

Total DNA was extracted from different isolates from each root sample using CTAB as described previously [34]. Using *Taq* polymerase (Fermentas), the ITS and LSU regions of nrDNA were amplified using ITS1F-ITS4 and LR0R-LR5 primer pairs, respectively, as described previously [35]. Sequencing of the amplicons was carried out with the primers used for the amplification by LGC GmbH (Berlin, Germany).

The sequences were compiled from electropherograms using the Pregap4 and Gap4 programs [36] and deposited into GenBank (JN859221–JN859491). The obtained sequences were compared to sequences in public databases using megablast (http://blast.ncbi.nlm.nih.gov/Blast.cgi) [37].

Multiple alignments of our sequences and datasets complemented by sequences from GenBank were made using MAFFT 6 Q-INS-i [38]. The alignments were checked and edited with ProSeq 2.9 [39]. The best-fit nucleotide substitution model was selected using the program jModelTest [40] considering the selection of Akaike Information Criterion (AIC). Maximum Likelihood (ML) phylogenetic analyses were carried out with the online version of PHYML 3.0 [41]. The GTR nucleotide substitution model was used with ML estimation of base frequencies. Six substitution-rate categories were set, and the gamma distribution parameter was estimated and optimized. Bootstrap analysis with 1000 replicates (MLB) was used to test the support of the branches. The same substitution model was used in Bayesian analyses performed with MrBayes 3.1.2 [42], [43]. Four Markov chains were run for 5,000,000 generations, sampling every 100 steps with a burn-in at 7500 sampled trees. To identify the main groups the ITS sequences of our isolates a Minimum Evolution tree was inferred using the Maximum Composite Likelihood model and pairwise deletion at gaps using MEGA 4.0 [44]. The phylogenetic trees were visualized and edited using the Tree Explorer of the MEGA4.0 program [44] and a text editor.

### In vitro tests

*In vitro* tests were performed with the representatives of each group by the analyses of ITS sequences of the fungal isolates (Fig. S2) using leek (*Allium porrum*) to test whether an isolate could be categorized as a DSE fungus. Seeds were surface-sterilized with 30% H_2_O_2_ for 1 min, then washed twice in sterile tap water for 10 min. Surface-sterilized seeds were germinated and watered on sterile filter paper for approximately seven days. Seedlings were laid to MS medium [45] (Sigma Basal Salt Mixture, 15 g/L agar, pH 5.8, 10 µg/ml Streptomycin) in 9-cm plastic Petri dishes. Two cuts (4–5 mm in diameter) of the representative fungal isolates were placed next to the roots of the leek. The plants were grown in a climatized growth chamber in a 14 h light (24°C)∶10 h dark (22°C) cycle. A total of five replicates for each fungal isolate and five control plants were incubated in each series. Plants were checked periodically and the roots were harvested 8–9 weeks after inoculation. We considered an isolate to be a DSE fungus if it colonized the root with no visible negative effect to the plant and formed microsclerotia in the roots.

### Microscopy

Root samples from the field samples and *in vitro* experiments were prepared for microscopic analyses similarly to the method described in Kovács and Bagi [21]. The cleared roots were stained using aniline blue and covered in PVLG (polyvinyl-lacto-glycerol). The samples were studied using light microscopes with Nomarski (differential interference contrast, DIC) optics and digital cameras.

## Results

### Fungal isolates

**Figure 1:**
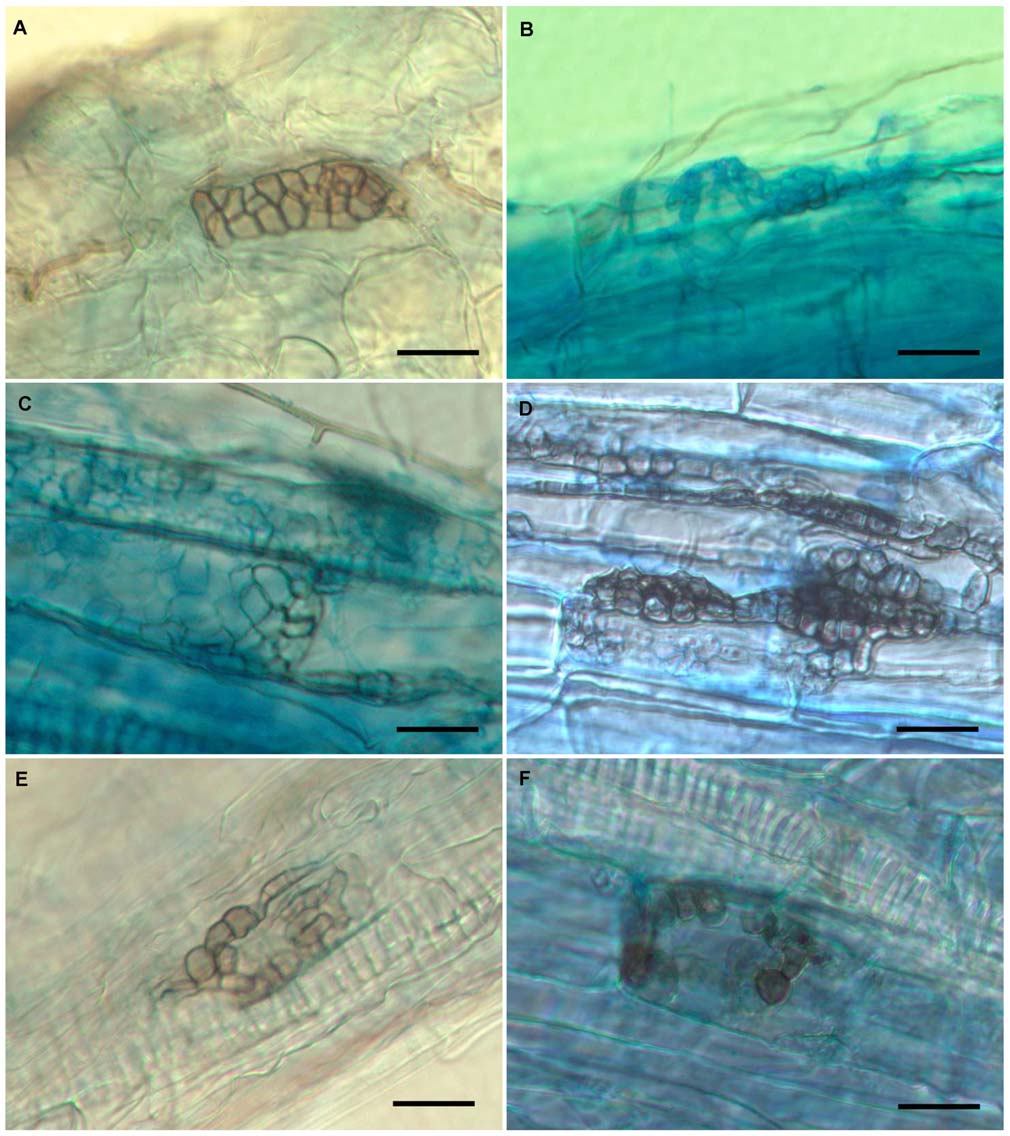
Microsclerotia formed by DSE fungi in roots. *Asclepias syriaca*, field collected sample (A). In artificial inoculation system with *Allium porrum* formed by REF025 (DSE-1 group) (B), REF096 (DSE-3 group) (C), REF101 (DSE-4 group) (D), REF132 (DSE-7 group) (E) and REF144 (DSE-8 group) (F). Bars = 20 µm.

All the field-collected root samples of the eleven plant species studied were colonized by intraradical melanized septate hyphae and microsclerotia (Fig. 1). In addition to these fungal structures, the roots were generally colonized by arbuscular mycorrhizal fungi.

**Figure 2:**
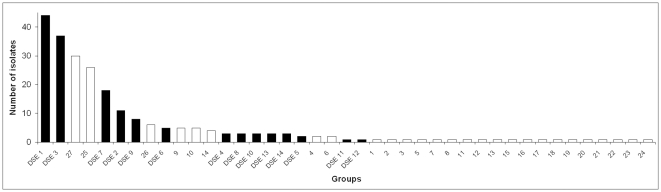
Frequency rank diagram of the 41 groups formed by the 241 fungal isolates. Filled columns represent the groups considered as DSE fungi.

Nearly 200 samples were collected from the eleven species from the three sampling areas (Table S1). In total, 296 fungal strains were isolated, and the ITS region of 241 isolates were sequenced and analyzed (Fig. S2). Based on analyses of the ITS sequences, the 241 isolates were grouped into 7 major and 34 smaller groups, including singletons (Fig. 2). The small groups with one or few isolates were found exclusively on either indigenous or invasive plants, but the groups with greater numbers of isolates colonized both invasive and indigenous plants. All but one of the isolates belonged to the phylum Ascomycota. The ITS of that one isolate showed similarities with sequences from the basidiomycete order Auriculariales (data not shown).

### Results of the in vitro inoculation tests

For the *in vitro* tests, 59 representative isolates of the 41 groups were chosen (Fig. S2). The healthy-looking *Allium porrum* plants generally had well-developed root systems and 3–4 leaves after 8–9 weeks. Nine isolates showed unambiguous negative effects on the hosts; the plants inoculated with these isolates died within 4–5 weeks, after their leaves and roots were shriveled and became yellow. In 21 cases, the leeks did not appear different from the controls. The fungi did not colonize the roots of *A. porrum*, and neither microsclerotia nor intraradical hyphae were observed. In tests of 29 isolates representing 14 groups, no negative effects were detected, and both septate endogenous hyphae and microsclerotia were observed in the colonized roots (Fig. 1). These 14 groups were considered DSE fungi.

The two most frequently isolated non-DSE groups were the widely distributed plant pathogens *Ilyonectria macrodidyma* (group 25) and *Fusarium oxysporum* (group 27). Both groups were isolated from several native and non-native plants from all sites (Fig. S2, Table S1).

### The DSE groups

**Figure 3:**
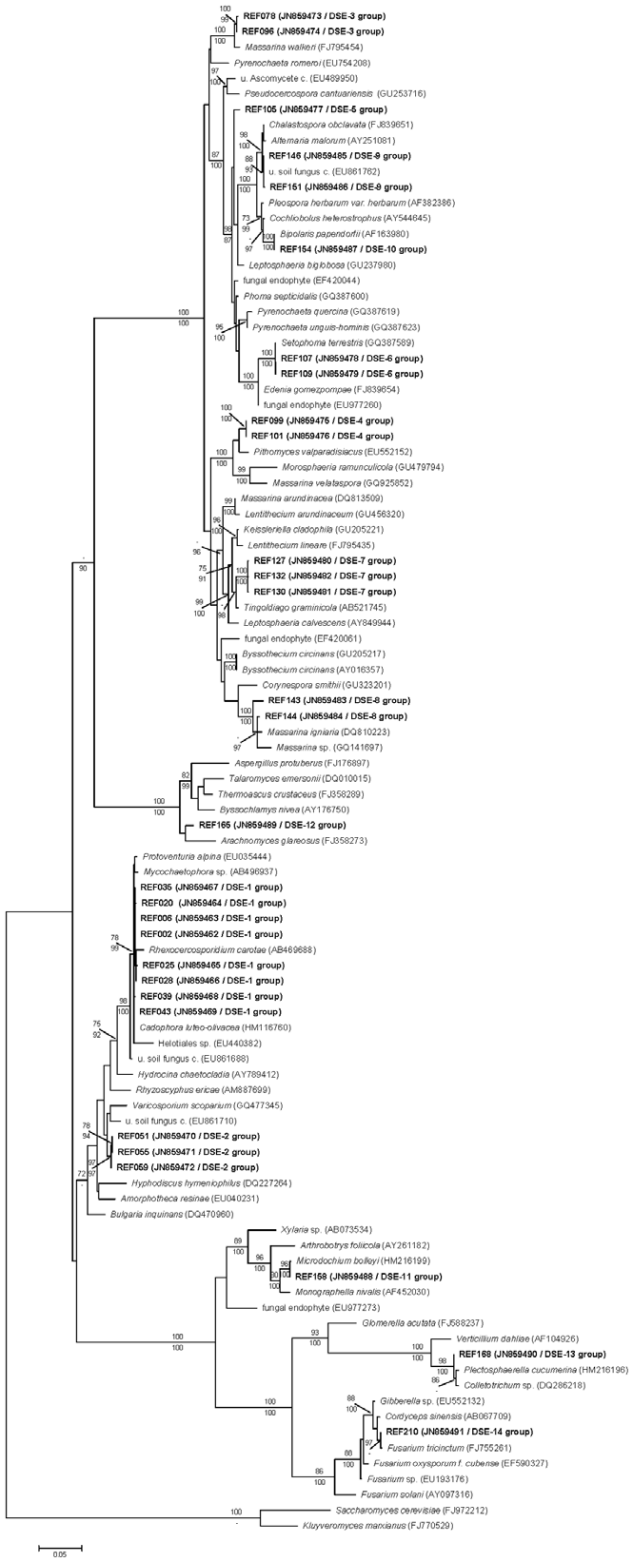
The maximum likelihood (ML) tree of the partial LSU sequences of representative isolates of DSE groups and similar sequences from GenBank. *Saccharomyces cerevisiae* (FJ972212) was used as outgroup. Sequences obtained in this study are shown in bold. Name of the DSE groups and accession number were shown in brackets. NJ bootstrap (not shown below 70%) values are above and the Bayesian posterior probabilities as percentage (not shown below 90%) are below the branches. Abbreviations: uncultured (u.), clone (c.). Bar = 0.05 expected change on one nucleotide.

Of the 41 groups of total isolates, 14 groups (∼35%) were considered DSE. These groups contained 142 of the 241 isolates (∼60%). Henceforth, these groups will be referred to as DSE groups 1–14 (Fig. 2, Fig. S2, Table S1). The analyses of ITS and LSU sequences showed no difference in the taxonomic position of the isolates. However, different representatives from public databases could be included in the two datasets, which resulted in complementary information about the DSE groups found in our sampling areas (Fig. 3, Table S2). All DSE isolates belonged to five orders of Ascomycota: Pleosporales (seven groups), Helotiales (two groups), Hypocreales (two groups), Eurotiales (one group), Xylariales (one group) and to the family Plectosphaerellaceae (one group) [46]. Some of the DSE groups could be identified at the species or genus level, whereas others could be identified only on higher taxonomic levels (Table S2). Neither the ITS or LSU sequences of some DSE groups (DSE-2, -4, -5, -12) showed similarity with any identified GenBank entry (Fig. 3, Table S2). The BLAST analysis of the ITS sequences of the DSE isolates showed high similarities with sequences of fungi from roots of different hosts or from soil originating from different geographic and climatic regions (Table S2).

Forty-eight isolates from the marked *Juniperus communis* trees of the three sampling sites were categorized as DSE fungi. All but one of the isolates belonged to DSE-1, -2 and -3 groups (Table S1). The DSE-1 group contains isolates originating from the three areas and three seasons. Isolates of the DSE-2 group represent two areas (Fülöpháza and Tatárszentgyörgy) and three seasons, and isolates from the DSE-3 group were obtained from two areas (Fülöpháza and Tatárszentgyörgy) and from two seasons (spring, autumn).

Accordingly, none of these three groups contained an isolate from only one area or one season. The fungi belonging to frequent DSE groups were isolated from both indigenous and invasive plants. In addition to the singleton groups (DSE-11, DSE-12), groups with few isolates (number of isolates indicated by slashes after the groups) originated only from either invasive (DSE-5/2/, DSE-13/3/ and DSE-14/3/) or native (DSE-4/3/ and DSE-8/3/) plant species (Fig. S2, Table S1). The DSE-9 contained more than 3 isolates: these 8 isolates originated solely from native plants from one site (Fülöpháza) during different seasons. Apart from the singletons, three DSE groups were from one season/sampling and one site (DSE-4/3/, DSE-5/3/ and DSE-8/3/). Finally, one group was from one season/sampling (DSE-10/3/) and two groups were isolated from only one site (DSE-6/5/ and DSE-9/8/).

## Discussion

Sandy areas in the Carpathian Basin of Danube-Tiscia interfluve, especially those of the open grasslands, represent semiarid regions with semidesert characteristics [26]. Our previous work on the root colonization of plants of sandy grassland in this region revealed the frequent presence of structures characteristic of DSE fungi [22]. Although our sampling in this study was not designed to address quantitative questions, the rank-order distribution of both the total 41 fungal groups isolated and the 14 groups categorized as DSE fungi showed similar distribution to those generally obtained in fungal endophyte studies [47]. Only a few fungal taxa are represented by the majority of the isolates, thus these groups could be categorized as dominant or at least common members of the RAF community of the area. Twenty-one groups were represented by only one isolate. The presence of ‘singletons’ and groups with low numbers of isolates can complicate the test of any specificity. However, a low isolation frequency does not necessarily mean low abundance; a low frequency could be the result of a biased isolation technique.

Studies using molecular diversity screening methods generally detect basidiomycetes, mostly Agaricales, as common members of RAF communities (e.g., [10], [18], [20]). We found only a single basidiomycete isolate with ITS showing similarities to the genus *Auricularia* (Auriculariales). Unfortunately, the isolate was lost before we could perform any experiments with the fungus.

Although there is an increasing interest in DSE fungi, there are no clearly defined criteria to determine whether a fungus is DSE. Such criteria would be useful in avoiding confusion caused by inconsequent terminology as has occurred in some mycorrhizal examples [48]. Several studies use a simple assumption: a fungus is ‘endophytic’ if it has been isolated from a healthy plant after surface sterilization [3]. In addition to the cultivation-based techniques, data arising from molecular diversity studies using fungal-specific primers are now used to characterize RAF and other endogenous fungal communities. In our study, we isolated fungi from surface-sterilized roots and used an artificial synthesis system to decide whether they may be categorized as DSE fungi. Based on our results, the majority of the most frequently isolates was DSE fungi. The DSE interactions, similarly to any plant-fungal interaction, depend on hosts and environmental factors [9]. Based on our artificial test, we may assume that each isolate that was categorized as DSE could be endophytic fungi. However, we cannot exclude the possibility that the isolates that did not fit our assumptions are DSE.

### The main DSE groups

The DSE isolates identified in this study belong to those ascomycete groups that are the most frequent root-colonizing endophytes, such as Pleosporales, Hypocreales and Helotiales.

Some of our isolates represented well-known root colonizing taxa or lineages. *Cadophora* formed the largest (44 isolates) DSE group (DSE-1); *Cadophora* is a widely distributed, common root colonizer, especially *C. finlandia* (syn: *Phialophora finlandia*) [49]. However, the species identity of our *Cadophora* group is dubious and it may represent more than one taxon (Fig. S3).

The group DSE-3 comprises *Rhizopycnis vagum* isolates. Several fungal root endophytes grouped together with our isolates from different geographic and host origin: such as the Mediterranean plants, *Pinus halepensis* and *Rosmarinus officinalis* [50], and *Dioscorea zingiberensis* from China [51] (Fig. S4, Table S2). Some studies characterize *R. vagum* as a pathogen. Armengol et al. [52] studied the pathogenicity of eight *R. vagum* isolates with different geographic and host origins. All isolates were pathogenic to muskmelon roots, although the disease reaction was not severe. *R. vagum* was also described as a weak pathogen and presumably a putative contributor to mature watermelon vine decline [53], [54]. There is a hypothesis stating that endophytic fungi might be latent pathogens [3]. The different effects of conspecific isolates reported in previous works might be the result of this, or there could be an unrevealed heterogeneity of the group masked by morphological and ITS sequence similarities.

Our DSE-8 group is the known DSE fungus *Periconia macrospinosa* (Fig. S5, Table S2). This species is widely distributed and is generally associated with grasses. The hyaline hyphae of the fungus are barely visible in the roots [55], except their microsclerotia, which are melanized. The colonization by this DSE fungus could remain hidden when roots are studied using routine methods. Three different *P. macrospinosa* isolates have been used in experimental studies of the effect of DSE fungal inoculation with several hosts [11], [19]. The results suggest a trend; namely, the grasses show stronger reactions to the inoculation with the species. We isolated this fungus only from grasses. This, together with the results of the aforementioned study, suggests that the fungus might be mainly associated with grasses.

Using ITS clone libraries of RAFs of *Bouteloua gracilis*, a dominant grass in a North American semiarid grassland, Porras-Alfaro et al. [18] found a dominant, unidentified fungal lineage belonging to Pleosporales. The lineage was detected in the RAF community of different grasses of semiarid grasslands (e.g., [10], [20], [56], [57]). In this study, we also found this particular fungal group: our group DSE-7 belongs to this clade. The group remains unidentified [18], and the analyses of our LSU sequences could not help identify the lineage either. At this time, this group has been detected in grass species. However, the previous studies detecting the lineage did not study non-grass plants. The majority of our isolates originated also from dominant grasses of our grasslands, such as *Festuca vaginata* and *Stypa borysthenica*, but the fungus was isolated from the roots of the dwarf-shrub *Fumana procumbens* and the invasive tree *Ailanthus altissima*. The isolates of the DSE-7 group could help us study the function and taxonomy of this DSE fungus that is distributed worldwide and seems to be associated with plants, especially with grasses, of (semi)arid habitats.

A mutual increase of heat tolerance was demonstrated when an endophytic *Curvularia* species was used in experiments with its natural host *Dichanthelium lanuginosum* originating from an area with strong geothermal activity [15]. The study showed not only the beneficial effect of the melanized fungus on the plant but also that the fungus needs the plant to survive the conditions of its natural habitat. In a later study, the same fungus was used with different host crops and yielded similar results [16]. Our DSE-10 group also belongs to the genus *Curvularia*. Based on the available sequence-similarity information given in the original article, our isolates are likely different from the fungus isolated by Redman et al. [15], on which a US patent on increasing heat tolerance using endophytic fungi is based (US Patent # 7906313).

*Microdochium* strains were isolated in prairie ecosystems of Kansas, United States, and the fungus was used in experimental studies about effects on plants and enzyme activities [11], [19]. The effect of the *Microdochium* varied according to different hosts, but the fungus is considered endophytic. The singleton group DSE-11 is a *Microdochium* showing high similarity with the isolate used in the aforementioned experiments [11], [19].

The DSE-14 group is composed of isolates of the widely distributed plant pathogen genus *Fusarium* that includes endophytic lineages [58]–[60]. RAF sequences represent fungi from grasslands of North America, and they also can be found in this group [10], [20], suggesting that the lineage could be widely spread.

### Specificity, seasonality, invasive plants

There are many results suggesting that DSE fungi are generalists and colonize several hosts [5]. However, only a few studies have addressed the area-, host- and organ-specificity and seasonality of these fungi. Herrera et al. [10] found only exceptional cases in which RAF could be detected in organs other than root. In the same study, in addition to some lineages showing geographically related specificity, a core of root-colonizing fungi was identified as constant members of a community of grass root endophytes. A similar pattern was found by Khidir et al. [20], who concluded that the dominant colonizers are similar in the American semiarid grasslands that they studied. We isolated specimens not only from grasses but also from other native and invasive plants of the area. Among the frequent groups there was only one, the group DSE-9 representing an *Embellisia* sp., that was isolated solely from native plants. The puzzling question of host specificity could be addressed on several levels [61]. Although we can conclude that the most frequent DSE fungi are generalists, more studies are necessary to obtain data from the individual to the ecosystem level. Mandyam et al. [11], [19] studied several *Periconia* isolates and concluded that DSE fungi have ‘broad host range’, but there was a considerable difference in the characteristics and effects of the conspecific isolates. Mandyam et al. [11] suggested a kind of ‘greater compatibility’ of the DSE isolates they studied with grasses than with other plants used in the experiments. It is relatively difficult to address whether there is a certain DSE fungus with specificity for grasses, as studies characterizing RAF and DSE communities are dominated with works focusing on grasses.

The seasonality of DSE fungi has been addressed in previous works ([62] and references therein). However, these works studied the seasonality of colonization by DSE fungi. Based on the results of our sampling in different seasons, the dominant members of the DSE community were not restricted to a certain period of the year. Nevertheless, understanding the seasonal dynamics, both functional and compositional, of DSE communities requires further studies.

In addition to the native plants, we studied the DSE fungi that colonized alien, invasive plants of the area. *Ailanthus altissima*, *Asclepias syriaca* and *Ambrosia artemisiifolia* are important invaders [63]–[65] causing not only ecological but economic problems as well. Each of the three species was found to be colonized by DSE fungi in the present work and in previous studies carried out in the region [21], [22]. The possible effects of soil microbiota on invasive plants and on the success of invasion is generally known, and the effects of mycorrhizal colonization have been frequently studied [29]. However, we are not aware of any study on the DSE fungi that colonize invasive plants. The DSE colonization of *Asclepias syriaca* and *Ambrosia artemisiifolia* were studied in their native environments in North America, and both were found to be colonized by DSE; the former was also used in experiments with *Periconia* isolates [11]. A successful invasive plant cannot depend exclusively on specific symbiotic partners, and we assume that the generalist root-colonizing fungi will colonize alien and invasive plants. In our study, the dominant, frequent DSE groups were isolated from the roots of the invasive plants as well as the native plants, which supports the hypothesis that those fungi are generalists with a wide host range. As the DSE fungi are frequent and their role in plant survival in a stressful environment could be important, we may assume that those fungi could also help invasive plants in these environments. Subsequent experiments are needed to study the effects of DSE fungi on the success of invasive plants.

### Conclusions

Based on the results of several studies, Khidir et al. [20] concluded that ‘grasses of the arid regions of North America share a general community of RAF’. Based on our own results, we may extend this statement by hypothesizing that plants of (semi)arid grasslands share common dominant DSE fungal community. Global, inter-continental comparative studies could test this hypothesis further. DSE fungi are frequently found in arid and semiarid environments, and these communities share some important global key-players. The need for a better understanding of the role of DSE fungi has been stressed by many authors (*e.g.*, [3], [12], [19]). The appropriate studies would require diversity screening, experimental studies and systems biology approaches [3]. All the dominant DSE groups identified in our study were generalists that colonized different native and invasive plants and showed no specificity to any area. Furthermore, they showed high similarity, even identity, with root colonizers from different continents; this strengthens the evidence that DSE fungi are generalists. Thus, our isolates from the semiarid sandy grasslands could be used in experiments to help understand the ‘elusive function’ [12] of DSE fungi in (semi)arid environments on a broader scale.

## Supporting Information

Figure S1 **The three sampling site on the Great Hungarian Plain; Bugac (a), Fülöpháza (b) and Tatárszentgyörgy (c).**
(PDF)

Figure S2 **Minimum Evolution tree of the ITS sequences of 241 isolates originating from roots of indigenous (O) and invasive (▴) plants.** Name of the groups is shown in brackets. Isolates used in the artificial inoculation experiments are marked (X). Tree was inferred using the Maximum Composite Likelihood model and pairwise deletion at gaps using MEGA 4.0. Bootstrap values obtained from 1000 replicates are shown as percentages and not shown below 70%. Bar = 2 changes/100 characters.
(PDF)

Figure S3 **The maximum likelihood (ML) tree of the ITS sequences of representatives of group DSE-1 and similar sequences from GenBank.** Sequences obtained in this study are shown in bold. *Pyrenopeziza revincta* (AJ4302224) was used as outgroup. Accession number, isolation source and geographic origin of sequences from public databases are shown. NJ bootstrap (not shown below 70%) values are above and the Bayesian posterior probabilities as percentage (not shown below 90%) are below the branches. Abbreviations: uncultured (u.), clone (c.). Bar = 0.05 expected change on one nucleotide.
(PDF)

Figure S4 **The maximum likelihood (ML) tree of the ITS sequences of representatives of group DSE-3 and similar sequences from GenBank.** Sequences obtained in this study are shown in bold. *Massarina corticola* (FR668004) was used as outgroup. Accession number, isolation source and geographic origin of sequences from public databases are shown. NJ bootstrap (not shown below 70%) values are above and the Bayesian posterior probabilities as percentage (not shown below 90%) are below the branches. Abbreviations: uncultured (u.), clone (c.). Bar = 0.1 expected change on one nucleotide.
(PDF)

Figure S5 **The maximum likelihood (ML) tree of the ITS sequences of representatives of group DSE-8 and similar sequences from GenBank.** Sequences obtained in this study are shown in bold. *Corollospora intermedia* (EU557363) was used as outgroup. Accession number, isolation source and geographic origin of sequences from public databases are shown. NJ bootstrap (not shown below 70%) values are above and the Bayesian posterior probabilities as percentage (not shown below 90%) are below the branches. Abbreviations: uncultured (u.), clone (c.). Bar = 0.1 expected change on one nucleotide.
(PDF)

Table S1 **The list of the 241 isolates with their name, group, accession number of ITS sequences, host plant, sampling site and season of the collection.**
(PDF)

Table S2 **Closest BLAST matches of representative isolates of DSE groups.** Name, source, provenance country, query coverage and max ident of sequences from GenBank are indicated. Abbreviations: uncultured (u.), clone (c.), strain (st.), Host (H), Isolation Source (IS), Tissue Type (TT), Environmental Sample (ES).
(PDF)

## References

[1] Schulz B, Boyle C (2005). The endophytic continuum.. Mycol Res.

[2] Saikkonen K, Faeth SH, Helander M, Sullivan TJ (1998). Fungal endophytes: A continuum of interactions with host plants.. Annu Rev Ecol Syst.

[3] Porras-Alfaro A, Bayman P (2011). Hidden fungi, emergent properties: Endophytes and microbiomes.. Annu Rev Phytopathol.

[4] Cheplick GP, Faeth SH (2009). Ecology and evolution of the grass-endophyte symbiosis.

[5] Jumpponen A, Trappe JM (1998). Dark septate endophytes: a review of facultative biotrophic root-colonizing fungi.. New Phytol.

[6] Yu T, Nassuth A, Peterson RL (2001). Characterization of the interaction between the dark septate fungus *Phialocephala fortini* and *Asparagus officinalis* roots.. Can J Microbiol.

[7] Narisawa K, Usuki F, Hashiba T (2004). Control of *Verticillium* yellows in Chinese cabbage by the dark septate endophytic fungus LtVB3.. Phytopathology.

[8] Addy HD, Piercey MM, Currah RS (2005). Microfungal endophytes in roots.. Can J Bot.

[9] Newsham KK (2011). A meta-analysis of plant responses to dark septate root endophytes.. New Phytol.

[10] Herrera J, Khidir HH, Eudy DM, Porras-Alfaro A, Natvig DO (2010). Shifting fungal endophyte communities colonize *Bouteloua gracilis*: effect of host tissue and geographical distribution.. Mycologia.

[11] Mandyam K, Fox C, Jumpponen A (2012). Septate endophyte colonization and host responses of grasses and forbs native to a tallgrass prairie.. Mycorrhiza.

[12] Mandyam K, Jumpponen A (2005). Seeking the elusive function of root-colonising dark septate endophytic fungi.. Stud Mycol.

[13] Read DJ, Haselwandter K (1981). Observations on the mycorrhizal status of some alpine plant communities.. New Phytol.

[14] Bell AA, Wheeler MH (1986). Biosynthesis and functions of fungal melanins.. Annu Rev Phytopathol.

[15] Redman RS, Sheehan KB, Stout RG, Rodriguez RJ, Henson JM (2002). Thermotolerance generated by plant/fungal symbiosis.. Science.

[16] Pennisi E (2003). Fungi shield new host plants from heat and drought.. Science.

[17] McLellan CA, Turbyville TJ, Wijeratne EMK, Kerschen A, Vierling E (2007). A rhizosphere fungus enhances *Arabidopsis* thermotolerance through production of an HSP90 inhibitor1.. Plant Physiol.

[18] Porras-Alfaro A, Herrera J, Sinsabaugh RL, Odenbach KJ, Lowrey T (2008). Novel root fungal consortium associated with a dominant desert grass.. Appl Environ Microbiol.

[19] Mandyam K, Loughin T, Jumpponen A (2010). Isolation and morphological and metabolic characterization of common endophytes in annually burned tallgrass prairie.. Mycologia.

[20] Khidir HH, Eudy DM, Porras-Alfaro A, Herrera J, Natvig DO (2010). A general suite of fungal endophytes dominate the roots of two dominant grasses in a semiarid grassland.. J Arid Environ.

[21] Kovács GM, Bagi I (2001). Mycorrhizal status of a mixed deciduous forest from the Great Hungarian Plain with special emphasis on the potential mycorrhizal partners of *Terfezia terfezioides* (Matt.) Trappe.. Phyton.

[22] Kovács GM, Szigetvári C (2002). Mycorrhizae and other root-associated fungal structures of the plants of a sandy grassland on the Great Hungarian Plain.. Phyton.

[23] Fekete G, Tórthmérész B (1993). Vegetation science in Hungary.. J Veg Sci.

[24] Kovács-Láng E, Molnár E, Kröel-Dulay G, Barabás S (2008). The KISKUN LTER: Long-term ecological research in the Kiskunság, Hungary.

[25] Borhidi A, Sánta A (1999). Vörös könyv Magyarország növénytársulásairól [Red Data Book of the Hungarian Plant Communities].

[26] Fekete G, Molnár Z, Kun A, Botta-Dukát Z (2002). On the structure of the Hungarian forest steppe: grasslands on sand.. Acta Zoo Acad Sci Hung.

[27] Richardson DM, Allsopp N, D'Antonio CM, Milton SJ, Rejmanek M (2000). Plant invasions - the role of mutualisms.. Biol Rev Camb Philos Soc.

[28] Callaway RM, Thelen GC, Rodriguez A, Holben WE (2004). Soil biota and exotic plant invasion.. Nature.

[29] Pringle A, Bever JD, Gardes M, Parrent JL, Rillig MC (2009). Mycorrhizal symbioses and plant invasions.. Annu Rev Ecol Evol Syst.

[30] Bever JD, Dickie IA, Facelli E, Facelli JM, Klironomos JN (2010). Rooting theories of plant ecology in microbial interactions.. Trends Ecol Evol.

[31] Várallyai Gy, Szujkó-Lacza J, Kováts D (1993). Soils in the region between the rivers Danube and Tisza (Hungary).. The Flora of the Kiskunság National Park.

[32] Kovács-Láng E, Kröel-Dulay Gy, Kertész M, Fekete G, Mika J (2000). Changes in the composition of sand grasslands along a climatic gradient in Hungary and implications for climate change.. Phytocoenologia.

[33] Marx DH (1969). The influence of ectotrophic mycorrhizal fungi on the resistance of pine roots to pathogenic infection. I. Antagonism of mycorrhizal fungi to root pathogenic infection fungi and soil bacteria.. Phytopathology.

[34] Jakucs E, Kovács GM, Agerer R, Romsics C, Erős-Honti Z (2005). Morphological-anatomical characterization and molecular identification of *Tomentella stuposa* ectomycorrhizae and related anatomotypes.. Mycorrhiza.

[35] Kovács GM, Trappe JM, Alsheikh AM, Bóka K, Elliott TF (2008). *Imaia*, a new truffle genus to accommodate *Terfezia gigantea*.. Mycologia.

[36] Staden R, Beal KF, Bonfield JK (2000). The Staden package, 1998.. Methods Mol Biol.

[37] Altschul SF, Gish W, Miller W, Myers EW, Lipman DJ (1990). Basic local alignment search tool.. J Mol Biol.

[38] Katoh K, Toh H (2008). Improved accuracy of multiple ncRNA alignment by incorporating structural information into a MAFFT-based framework.. BMC Bioinformatics.

[39] Filatov DA (2002). ProSeq: a software for preparation and evolutionary analysis of DNA sequence data sets.. Mol Ecol Notes.

[40] Posada D (2008). jModelTest: Phylogenetic Model Averaging.. Mol Biol Evol.

[41] Guindon S, Dufayard JF, Lefort V, Anisimova M, Hordijk W (2010). New Algorithms and Methods to Estimate Maximum-Likelihood Phylogenies: Assessing the Performance of PhyML 3.0.. Syst Biol.

[42] Huelsenbeck JP, Ronquist F (2001). MRBAYES: Bayesian inference of phylogenetic trees.. Bioinformatics.

[43] Ronquist F, Huelsenbeck JP (2003). MrBayes 3: Bayesian phylogenetic inference under mixed models.. Bioinformatics.

[44] Tamura K, Dudley J, Nei M, Kumar S (2007). MEGA4: Molecular Evolutionary Genetics Analysis (MEGA) software version 4.0.. Mol Biol Evol.

[45] Murashige T, Skoog F (1962). A revised medium for rapid growth and bioassays with tobacco tissue cultures.. Physiol Plant.

[46] Réblová M, Gams W, Seifert KA (2011). *Monilochaetes* and allied genera of the *Glomerellales*, and a reconsideration of families in the *Microascales*.. Stud Mycol.

[47] Sánchez Márquez S, Bills GF, Domínguez Acuña L, Zabalgogeazcoa I (2010). Endophytic mycobiota of leaves and roots of the grass *Holcus lanatus*.. Fungal Divers.

[48] Brundrett MC (2009). Mycorrhizal associations and other means of nutrition of vascular plants: understanding the global diversity of host plants by resolving conflicting information and developing reliable means of diagnosis.. Plant Soil.

[49] Harrington TC, McNew D (2003). Phylogenetic analysis places the *Phialophora*-like anamorph genus *Cadophora* in the Helotiales.. Mycotaxon.

[50] Girlanda M, Ghignone S, Luppi AM (2002). Diversity of sterile root-associated fungi of two mediterranean plants.. New Phytol.

[51] Xu L, Zhou L, Zhao J, Li J, Li X, Wang J (2008). Fungal endophytes from *Dioscorea zingiberensis* rhizomes and their antibacterial activity.. Lett Appl Microbiol.

[52] Armengol J, Vicent A, Martínez-Culebras P, Bruton BD, García-Jiménez J (2003). Identification, occurrence and pathogenicityy of *Rhizopycnis vagum* on muskmelon in Spain.. Plant Pathol.

[53] Farr DF, Miller ME, Bruton BD (1998). *Rhizopycnis vagum* gen. et sp. nov., a new coelomycetous fungus from roots of melons and sugarcane.. Mycologia.

[54] Westphal A, Xing L, Goodwin SB (2011). Mature watermelon vine decline: Suppression with fumigants of a soil-borne problem and association with *Rhizopycnis vagum*.. Crop Prot.

[55] Barrow JR, Aaltonen RE (2001). Evaluation of the internal colonization of *Atriplex canescens* (Pursh) Nutt. roots by dark septate fungi and the influence of host physiological activity.. Mycorrhiza.

[56] Hawkes CV, Belnap J, D'Antonio C, Firestone MK (2006). Arbuscular mycorrhizal assemblages in native plant roots change in the presence of invasive exotic grasses.. Plant Soil.

[57] Porras-Alfaro A, Herrera J, Natvig DO, Lipinski K, Sinsabaugh RL (2011). Diversity and distribution patterns of soil fungal communities in a semiarid grassland.. Mycologia.

[58] Phan HT, Burgess LW, Summerell BA, Bullock S, Liew ECY (2004). *Gibberella gaditjirrii* (*Fusarium gaditjirrii*) sp. nov., a new species from tropical grasses in Australia.. Stud Mycol.

[59] Paparu P, Dubois T, Gold C, Niere B, Adipala E (2006). Colonisation pattern of nonpathogenic *Fusarium oxysporum*, a potential biological control agent, in roots and rhizomes of tissue cultured *Musa* plantlets.. An Appl Biol.

[60] Zhan JX, Burns AM, Liu MPX, Faeth SH, Gunatilaka AAL (2007). Search for cell motility and angiogenesis inhibitors with potential anticancer activity: Beauvericin and other constituents of two endophytic strains of *Fusarium oxysporum*.. J Nat Prod.

[61] Poulin R, Krasnov BR, Mouillot D (2011). Host specificity in phylogenetic and geographic space.. Trends Parasitol.

[62] Mandyam K, Jumpponen A (2008). Seasonal and temporal dynamics os arbuscular mycorrhizal and dark septate endophytic fungi in a tallgrass prairie ecosystem are minimally affected by nitrogen enrichment.. Mycorrhiza.

[63] Bagi I, Mihály B, Botta-Dukát Z (2004). Selyemkóró.. Biológiai inváziók Magyarországon. Özönnövények.

[64] Udvardy L, Mihály B, Botta-Dukát Z (2004). Bálványfa.. Biológiai inváziók Magyarországon. Özönnövények.

[65] Szigetvári C, Benkő Z, Mihály B, Botta-Dukát Z (2004). Parlagfű.. Biológiai inváziók Magyarországon. Özönnövények.

