# Supplementary material for: The Dark Side Is Not Fastidious – Dark Septate Endophytic Fungi of Native and Invasive Plants of Semiarid Sandy Areas
Source: PLoS One. 2012 Feb 29;7(2):e32570. doi: 10.1371/journal.pone.0032570 (PMC3290574; doi:10.1371/journal.pone.0032570)

Figure S1 The three sampling site on the Great Hungarian Plain; Bugac (A), Fülöpháza (B) and Tatárszentgyörgy (C).

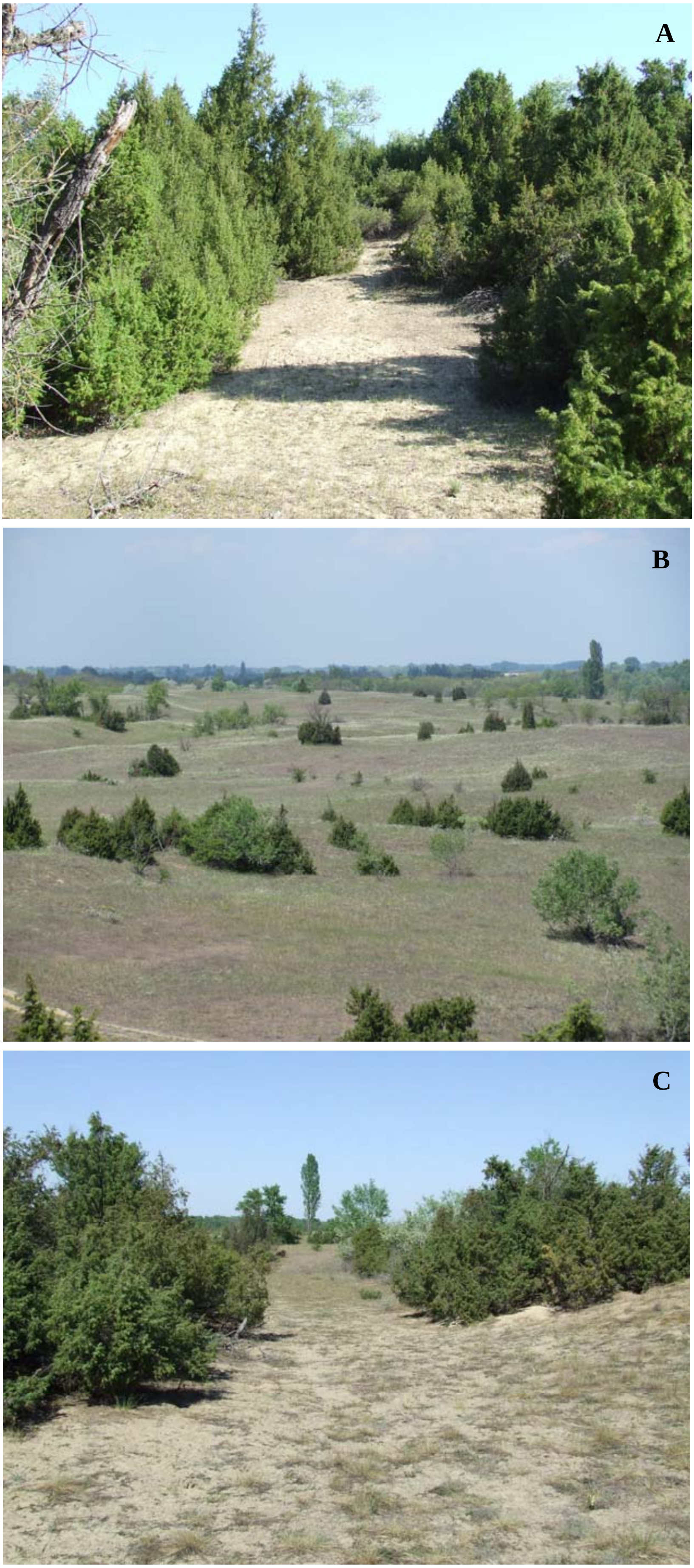

Supplement: Figure S1 — The three sampling site on the Great Hungarian Plain; Bugac (a), Fülöpháza (b) and Tatárszentgyörgy (c). (PDF) [file pone.0032570.s001.pdf]
