# Supplementary material for: The Dark Side Is Not Fastidious – Dark Septate Endophytic Fungi of Native and Invasive Plants of Semiarid Sandy Areas
Source: PLoS One. 2012 Feb 29;7(2):e32570. doi: 10.1371/journal.pone.0032570 (PMC3290574; doi:10.1371/journal.pone.0032570)
Supplement: Figure S2 — Minimum Evolution tree of the ITS sequences of 241 isolates originating from roots of indigenous (O) and invasive (▴) plants. Name of the groups is shown in brackets. Isolates used in the artificial inoculation experiments are marked (X). Tree was inferred using the Maximum Composite Likelihood model and pairwise deletion at gaps using MEGA 4.0. Bootstrap values obtained from 1000 replicates are shown as percentages and not shown below 70%. Bar = 2 changes/100 characters. (PDF) [file pone.0032570.s002.pdf]

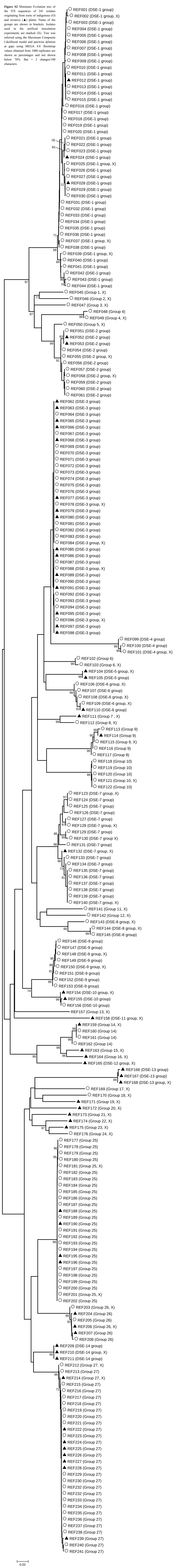

Figure S2 Minimum Evolution tree of the ITS sequences of 241 isolates originating from roots of indigenous (O) and invasive (▲) plants. Name of the groups are shown in brackets. Isolates used in the artificial inoculation experiments are marked (X). Tree was inferred using the Maximum Composite Likelihood model and pairwise deletion at gaps using MEGA 4.0. Bootstrap values obtained from 1000 replicates are shown as percentages and not shown below 70%. Bar = 2 changes/100 characters.
