# Supplementary material for: The Dark Side Is Not Fastidious – Dark Septate Endophytic Fungi of Native and Invasive Plants of Semiarid Sandy Areas
Source: PLoS One. 2012 Feb 29;7(2):e32570. doi: 10.1371/journal.pone.0032570 (PMC3290574; doi:10.1371/journal.pone.0032570)
Supplement: Figure S5 — The maximum likelihood (ML) tree of the ITS sequences of representatives of group DSE-8 and similar sequences from GenBank. Sequences obtained in this study are shown in bold. Corollospora intermedia (EU557363) was used as outgroup. Accession number, isolation source and geographic origin of sequences from public databases are shown. NJ bootstrap (not shown below 70%) values are above and the Bayesian posterior probabilities as percentage (not shown below 90%) are below the branches. Abbreviations: uncultured (u.), clone (c.). Bar = 0.1 expected change on one nucleotide. (PDF) [file pone.0032570.s005.pdf]

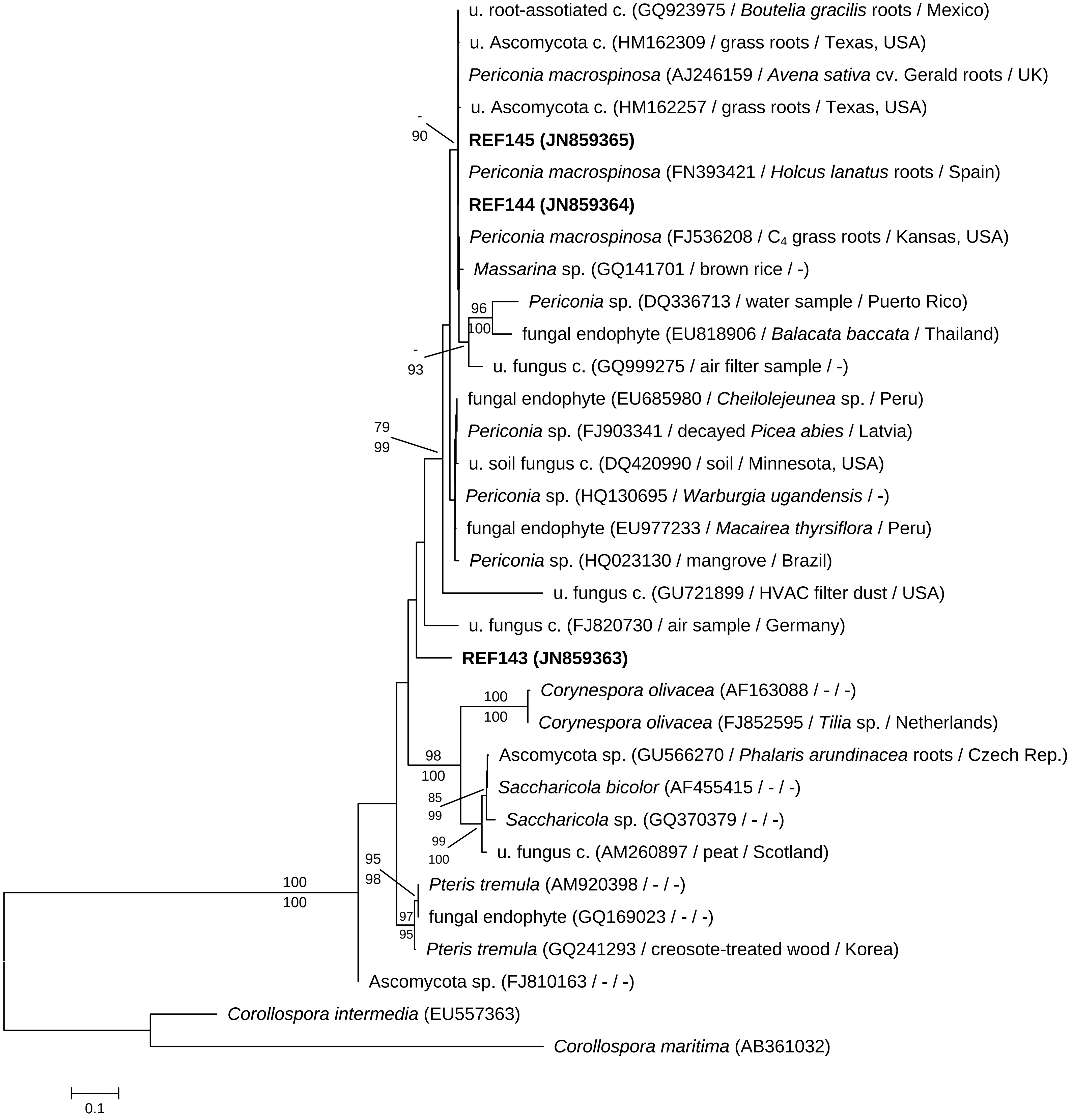

Figure S5 The maximum likelihood (ML) tree of the ITS sequences of representatives of group DSE-8 and similar sequences from GenBank. Sequences obtained in this study are shown in bold. *Corollospora intermedia* (EU557363) was used as outgroup. Accession number, isolation source and geographic origin of sequences from public databases are shown. NJ bootstrap (not shown below 70%) values are above and the Bayesian posterior probabilities as percentage (not shown below 90%) are below the branches. Abbreviations: uncultured (u.), clone (c.). Bar = 0.1 expected change on one nucleotide.
