# Supplementary material for: The Dark Side Is Not Fastidious – Dark Septate Endophytic Fungi of Native and Invasive Plants of Semiarid Sandy Areas
Source: PLoS One. 2012 Feb 29;7(2):e32570. doi: 10.1371/journal.pone.0032570 (PMC3290574; doi:10.1371/journal.pone.0032570)
Supplement: Table S1 — The list of the 241 isolates with their name, group, accession number of ITS sequences, host plant, sampling site and season of the collection. (PDF) [file pone.0032570.s006.pdf]

**Table S1** The list of the 241 isolates with their name, group, accession number of ITS sequences, host plant, sampling site and season of the collection.

| Isolate | Group | Accession | Host | Site | Season |
|---|---|---|---|---|---|
| REF001 | DSE-1 | JN859221 | *Juniperus communis* | Bugac | Summer |
| REF002 | DSE-1 | JN859222 | *Juniperus communis* | Tatárszentgyörgy | Spring |
| REF003 | DSE-1 | JN859223 | *Helianthemum ovatum* | Fülöpháza | Summer |
| REF004 | DSE-1 | JN859224 | *Juniperus communis* | Bugac | Summer |
| REF005 | DSE-1 | JN859225 | *Juniperus communis* | Bugac | Summer |
| REF006 | DSE-1 | JN859226 | *Juniperus communis* | Bugac | Summer |
| REF007 | DSE-1 | JN859227 | *Juniperus communis* | Tatárszentgyörgy | Summer |
| REF008 | DSE-1 | JN859228 | *Juniperus communis* | Bugac | Fall |
| REF009 | DSE-1 | JN859229 | *Juniperus communis* | Fülöpháza | Fall |
| REF010 | DSE-1 | JN859230 | *Juniperus communis* | Tatárszentgyörgy | Spring |
| REF011 | DSE-1 | JN859231 | *Juniperus communis* | Fülöpháza | Summer |
| REF012 | DSE-1 | JN859232 | *Ailanthus altissima* | Fülöpháza | Summer |
| REF013 | DSE-1 | JN859233 | *Helianthemum ovatum* | Fülöpháza | Summer |
| REF014 | DSE-1 | JN859234 | *Juniperus communis* | Tatárszentgyörgy | Fall |
| REF015 | DSE-1 | JN859235 | *Juniperus communis* | Fülöpháza | Fall |
| REF016 | DSE-1 | JN859236 | *Juniperus communis* | Tatárszentgyörgy | Spring |
| REF017 | DSE-1 | JN859237 | *Juniperus communis* | Fülöpháza | Spring |
| REF018 | DSE-1 | JN859238 | *Juniperus communis* | Fülöpháza | Spring |
| REF019 | DSE-1 | JN859239 | *Juniperus communis* | Bugac | Spring |
| REF020 | DSE-1 | JN859240 | *Juniperus communis* | Fülöpháza | Spring |
| REF021 | DSE-1 | JN859241 | *Juniperus communis* | Bugac | Fall |
| REF022 | DSE-1 | JN859242 | *Juniperus communis* | Tatárszentgyörgy | Fall |
| REF023 | DSE-1 | JN859243 | *Juniperus communis* | Fülöpháza | Summer |
| REF024 | DSE-1 | JN859244 | *Ailanthus altissima* | Fülöpháza | Summer |
| REF025 | DSE-1 | JN859245 | *Juniperus communis* | Bugac | Fall |
| REF026 | DSE-1 | JN859246 | *Juniperus communis* | Tatárszentgyörgy | Summer |
| REF027 | DSE-1 | JN859247 | *Juniperus communis* | Tatárszentgyörgy | Summer |
| REF028 | DSE-1 | JN859248 | *Asclepias syriaca* | Fülöpháza | Summer |
| REF029 | DSE-1 | JN859249 | *Juniperus communis* | Fülöpháza | Fall |
| REF030 | DSE-1 | JN859250 | *Juniperus communis* | Fülöpháza | Fall |
| REF031 | DSE-1 | JN859251 | *Juniperus communis* | Bugac | Spring |
| REF032 | DSE-1 | JN859252 | *Juniperus communis* | Bugac | Spring |
| REF033 | DSE-1 | JN859253 | *Juniperus communis* | Bugac | Spring |
| REF034 | DSE-1 | JN859254 | *Juniperus communis* | Fülöpháza | Spring |
| REF035 | DSE-1 | JN859255 | *Juniperus communis* | Bugac | Spring |
| REF036 | DSE-1 | JN859256 | *Juniperus communis* | Fülöpháza | Spring |
| REF037 | DSE-1 | JN859257 | *Populus alba* | Fülöpháza | Summer |
| REF038 | DSE-1 | JN859258 | *Juniperus communis* | Fülöpháza | Spring |
| REF039 | DSE-1 | JN859259 | *Juniperus communis* | Fülöpháza | Spring |
| REF040 | DSE-1 | JN859260 | *Juniperus communis* | Fülöpháza | Spring |
| REF041 | DSE-1 | JN859261 | *Juniperus communis* | Fülöpháza | Spring |
| REF042 | DSE-1 | JN859262 | *Populus alba* | Fülöpháza | Summer |
| REF043 | DSE-1 | JN859263 | *Juniperus communis* | Fülöpháza | Summer |
| REF044 | DSE-1 | JN859264 | *Juniperus communis* | Fülöpháza | Summer |
| REF045 | 1 | JN859265 | *Juniperus communis* | Fülöpháza | Spring |
| REF046 | 2 | JN859266 | *Juniperus communis* | Tatárszentgyörgy | Spring |
| REF047 | 3 | JN859267 | *Helianthemum ovatum* | Fülöpháza | Summer |
| REF048 | 4 | JN859268 | *Juniperus communis* | Bugac | Fall |
| REF049 | 4 | JN859269 | *Juniperus communis* | Tatárszentgyörgy | Spring |
| REF050 | 5 | JN859270 | *Juniperus communis* | Bugac | Spring |

| | | | | | |
|---|---|---|---|---|---|
| REF051 | DSE-2 | JN859271 | *Festuca procumbens* | Fülöpháza | Summer |
| REF052 | DSE-2 | JN859272 | *Asclepias syriaca* | Fülöpháza | Summer |
| REF053 | DSE-2 | JN859273 | *Ailanthus altissima* | Tatárszentgyörgy | Summer |
| REF054 | DSE-2 | JN859274 | *Helianthemum ovatum* | Fülöpháza | Spring |
| REF055 | DSE-2 | JN859275 | *Juniperus communis* | Tatárszentgyörgy | Spring |
| REF056 | DSE-2 | JN859276 | *Juniperus communis* | Fülöpháza | Spring |
| REF057 | DSE-2 | JN859277 | *Juniperus communis* | Fülöpháza | Summer |
| REF058 | DSE-2 | JN859278 | *Juniperus communis* | Tatárszentgyörgy | Summer |
| REF059 | DSE-2 | JN859279 | *Juniperus communis* | Fülöpháza | Summer |
| REF060 | DSE-2 | JN859280 | *Juniperus communis* | Fülöpháza | Fall |
| REF061 | DSE-2 | JN859281 | *Festuca procumbens* | Fülöpháza | Summer |
| REF062 | DSE-3 | JN859282 | *Ailanthus altissima* | Fülöpháza | Summer |
| REF063 | DSE-3 | JN859283 | *Ailanthus altissima* | Fülöpháza | Summer |
| REF064 | DSE-3 | JN859284 | *Populus alba* | Fülöpháza | Summer |
| REF065 | DSE-3 | JN859285 | *Asclepias syriaca* | Fülöpháza | Summer |
| REF066 | DSE-3 | JN859286 | *Ambrosia artemisiifolia* | Fülöpháza | Summer |
| REF067 | DSE-3 | JN859287 | *Festuca procumbens* | Fülöpháza | Summer |
| REF068 | DSE-3 | JN859288 | *Ailanthus altissima* | Fülöpháza | Summer |
| REF069 | DSE-3 | JN859289 | *Juniperus communis* | Tatárszentgyörgy | Fall |
| REF070 | DSE-3 | JN859290 | *Juniperus communis* | Fülöpháza | Spring |
| REF071 | DSE-3 | JN859291 | *Ephedra distachya* | Fülöpháza | Fall |
| REF072 | DSE-3 | JN859292 | *Ephedra distachya* | Fülöpháza | Fall |
| REF073 | DSE-3 | JN859293 | *Medicago minima* | Fülöpháza | Summer |
| REF074 | DSE-3 | JN859294 | *Medicago minima* | Fülöpháza | Summer |
| REF075 | DSE-3 | JN859295 | *Medicago minima* | Fülöpháza | Summer |
| REF076 | DSE-3 | JN859296 | *Medicago minima* | Fülöpháza | Summer |
| REF077 | DSE-3 | JN859297 | *Ailanthus altissima* | Tatárszentgyörgy | Summer |
| REF078 | DSE-3 | JN859298 | *Medicago minima* | Fülöpháza | Summer |
| REF079 | DSE-3 | JN859299 | *Ambrosia artemisiifolia* | Fülöpháza | Summer |
| REF080 | DSE-3 | JN859300 | *Ailanthus altissima* | Fülöpháza | Summer |
| REF081 | DSE-3 | JN859301 | *Medicago minima* | Fülöpháza | Summer |
| REF082 | DSE-3 | JN859302 | *Medicago minima* | Fülöpháza | Summer |
| REF083 | DSE-3 | JN859303 | *Populus alba* | Fülöpháza | Summer |
| REF084 | DSE-3 | JN859304 | *Ephedra distachya* | Fülöpháza | Fall |
| REF085 | DSE-3 | JN859305 | *Ailanthus altissima* | Tatárszentgyörgy | Fall |
| REF086 | DSE-3 | JN859306 | *Ambrosia artemisiifolia* | Tatárszentgyörgy | Fall |
| REF087 | DSE-3 | JN859307 | *Juniperus communis* | Fülöpháza | Fall |
| REF088 | DSE-3 | JN859308 | *Medicago minima* | Fülöpháza | Summer |
| REF089 | DSE-3 | JN859309 | *Ambrosia artemisiifolia* | Fülöpháza | Summer |
| REF090 | DSE-3 | JN859310 | *Medicago minima* | Fülöpháza | Summer |
| REF091 | DSE-3 | JN859311 | *Ambrosia artemisiifolia* | Tatárszentgyörgy | Summer |
| REF092 | DSE-3 | JN859312 | *Medicago minima* | Fülöpháza | Summer |
| REF093 | DSE-3 | JN859313 | *Ephedra distachya* | Fülöpháza | Fall |
| REF094 | DSE-3 | JN859314 | *Ephedra distachya* | Fülöpháza | Fall |
| REF095 | DSE-3 | JN859315 | *Ailanthus altissima* | Tatárszentgyörgy | Summer |
| REF096 | DSE-3 | JN859316 | *Juniperus communis* | Fülöpháza | Spring |
| REF097 | DSE-3 | JN859317 | *Ailanthus altissima* | Tatárszentgyörgy | Summer |
| REF098 | DSE-3 | JN859318 | *Ambrosia artemisiifolia* | Tatárszentgyörgy | Summer |
| REF099 | DSE-4 | JN859319 | *Populus alba* | Fülöpháza | Summer |
| REF100 | DSE-4 | JN859320 | *Populus alba* | Fülöpháza | Summer |
| REF101 | DSE-4 | JN859321 | *Populus alba* | Fülöpháza | Summer |
| REF102 | 6 | JN859322 | *Juniperus communis* | Bugac | Summer |
| REF103 | 6 | JN859323 | *Juniperus communis* | Bugac | Summer |
| REF104 | DSE-5 | JN859324 | *Ambrosia artemisiifolia* | Tatárszentgyörgy | Fall |
| REF105 | DSE-5 | JN859325 | *Ambrosia artemisiifolia* | Tatárszentgyörgy | Fall |
| REF106 | DSE-6 | JN859326 | *Ephedra distachya* | Fülöpháza | Fall |

| | | | | | |
|---|---|---|---|---|---|
| REF107 | DSE-6 | JN859327 | *Medicago minima* | Fülöpháza | Summer |
| REF108 | DSE-6 | JN859328 | *Ephedra distachya* | Fülöpháza | Fall |
| REF109 | DSE-6 | JN859329 | *Medicago minima* | Fülöpháza | Summer |
| REF110 | DSE-6 | JN859330 | *Ailanthus altissima* | Fülöpháza | Summer |
| REF111 | 7 | JN859331 | *Ailanthus altissima* | Tatárszentgyörgy | Summer |
| REF112 | 8 | JN859332 | *Populus alba* | Fülöpháza | Summer |
| REF113 | 9 | JN859333 | *Juniperus communis* | Tatárszentgyörgy | Summer |
| REF114 | 9 | JN859334 | *Ailanthus altissima* | Fülöpháza | Summer |
| REF115 | 9 | JN859335 | *Juniperus communis* | Tatárszentgyörgy | Spring |
| REF116 | 9 | JN859336 | *Juniperus communis* | Fülöpháza | Spring |
| REF117 | 9 | JN859337 | *Juniperus communis* | Fülöpháza | Spring |
| REF118 | 10 | JN859338 | *Helianthemum ovatum* | Fülöpháza | Spring |
| REF119 | 10 | JN859339 | *Helianthemum ovatum* | Fülöpháza | Spring |
| REF120 | 10 | JN859340 | *Festuca procumbens* | Fülöpháza | Spring |
| REF121 | 10 | JN859341 | *Festuca procumbens* | Fülöpháza | Spring |
| REF122 | 10 | JN859342 | *Festuca procumbens* | Fülöpháza | Spring |
| REF123 | DSE-7 | JN859343 | *Festuca vaginata* | Fülöpháza | Summer |
| REF124 | DSE-7 | JN859344 | *Festuca vaginata* | Fülöpháza | Summer |
| REF125 | DSE-7 | JN859345 | *Festuca vaginata* | Fülöpháza | Summer |
| REF126 | DSE-7 | JN859346 | *Festuca vaginata* | Fülöpháza | Summer |
| REF127 | DSE-7 | JN859347 | *Festuca vaginata* | Fülöpháza | Summer |
| REF128 | DSE-7 | JN859348 | *Festuca vaginata* | Fülöpháza | Summer |
| REF129 | DSE-7 | JN859349 | *Festuca vaginata* | Fülöpháza | Summer |
| REF130 | DSE-7 | JN859350 | *Festuca procumbens* | Fülöpháza | Summer |
| REF131 | DSE-7 | JN859351 | *Festuca vaginata* | Fülöpháza | Summer |
| REF132 | DSE-7 | JN859352 | *Ailanthus altissima* | Tatárszentgyörgy | Summer |
| REF133 | DSE-7 | JN859353 | *Festuca vaginata* | Fülöpháza | Summer |
| REF134 | DSE-7 | JN859354 | *Festuca vaginata* | Fülöpháza | Summer |
| REF135 | DSE-7 | JN859355 | *Stipa borysthenica* | Fülöpháza | Spring |
| REF136 | DSE-7 | JN859356 | *Festuca vaginata* | Fülöpháza | Summer |
| REF137 | DSE-7 | JN859357 | *Stipa borysthenica* | Fülöpháza | Spring |
| REF138 | DSE-7 | JN859358 | *Festuca vaginata* | Fülöpháza | Summer |
| REF139 | DSE-7 | JN859359 | *Festuca vaginata* | Fülöpháza | Summer |
| REF140 | DSE-7 | JN859360 | *Stipa borysthenica* | Fülöpháza | Spring |
| REF141 | 11 | JN859361 | *Festuca vaginata* | Fülöpháza | Summer |
| REF142 | 12 | JN859362 | *Juniperus communis* | Bugac | Fall |
| REF143 | DSE-8 | JN859363 | *Festuca vaginata* | Fülöpháza | Summer |
| REF144 | DSE-8 | JN859364 | *Festuca vaginata* | Fülöpháza | Summer |
| REF145 | DSE-8 | JN859365 | *Festuca vaginata* | Fülöpháza | Summer |
| REF146 | DSE-9 | JN859366 | *Medicago minima* | Fülöpháza | Summer |
| REF147 | DSE-9 | JN859367 | *Festuca procumbens* | Fülöpháza | Summer |
| REF148 | DSE-9 | JN859368 | *Medicago minima* | Fülöpháza | Summer |
| REF149 | DSE-9 | JN859369 | *Festuca procumbens* | Fülöpháza | Summer |
| REF150 | DSE-9 | JN859370 | *Festuca vaginata* | Fülöpháza | Summer |
| REF151 | DSE-9 | JN859371 | *Festuca vaginata* | Fülöpháza | Summer |
| REF152 | DSE-9 | JN859372 | *Festuca vaginata* | Fülöpháza | Summer |
| REF153 | DSE-9 | JN859373 | *Festuca vaginata* | Fülöpháza | Summer |
| REF154 | DSE-10 | JN859374 | *Ailanthus altissima* | Tatárszentgyörgy | Summer |
| REF155 | DSE-10 | JN859375 | *Ailanthus altissima* | Fülöpháza | Summer |
| REF156 | DSE-10 | JN859376 | *Juniperus communis* | Tatárszentgyörgy | Summer |
| REF157 | 13 | JN859377 | *Festuca procumbens* | Fülöpháza | Summer |
| REF158 | DSE-11 | JN859378 | *Ailanthus altissima* | Tatárszentgyörgy | Fall |
| REF159 | 14 | JN859379 | *Ailanthus altissima* | Fülöpháza | Summer |
| REF160 | 14 | JN859380 | *Juniperus communis* | Fülöpháza | Summer |
| REF161 | 14 | JN859381 | *Juniperus communis* | Bugac | Fall |
| REF162 | 14 | JN859382 | *Juniperus communis* | Bugac | Spring |

| | | | | | |
|---|---|---|---|---|---|
| REF163 | 15 | JN859383 | *Ailanthus altissima* | Fülöpháza | Summer |
| REF164 | 16 | JN859384 | *Ailanthus altissima* | Tatárszentgyörgy | Summer |
| REF165 | DSE-12 | JN859385 | *Ailanthus altissima* | Fülöpháza | Summer |
| REF166 | DSE-13 | JN859386 | *Ailanthus altissima* | Tatárszentgyörgy | Summer |
| REF167 | DSE-13 | JN859387 | *Ailanthus altissima* | Tatárszentgyörgy | Fall |
| REF168 | DSE-13 | JN859388 | *Asclepias syriaca* | Fülöpháza | Summer |
| REF169 | 17 | JN859389 | *Juniperus communis* | Fülöpháza | Spring |
| REF170 | 18 | JN859390 | *Juniperus communis* | Bugac | Spring |
| REF171 | 19 | JN859391 | *Ailanthus altissima* | Fülöpháza | Summer |
| REF172 | 20 | JN859392 | *Ambrosia artemisiifolia* | Tatárszentgyörgy | Fall |
| REF173 | 21 | JN859393 | *Asclepias syriaca* | Fülöpháza | Summer |
| REF174 | 22 | JN859394 | *Ailanthus altissima* | Tatárszentgyörgy | Summer |
| REF175 | 23 | JN859395 | *Ailanthus altissima* | Tatárszentgyörgy | Fall |
| REF176 | 24 | JN859396 | *Juniperus communis* | Tatárszentgyörgy | Spring |
| REF177 | 25 | JN859397 | *Juniperus communis* | Tatárszentgyörgy | Summer |
| REF178 | 25 | JN859398 | *Juniperus communis* | Tatárszentgyörgy | Summer |
| REF179 | 25 | JN859399 | *Juniperus communis* | Tatárszentgyörgy | Summer |
| REF180 | 25 | JN859400 | *Juniperus communis* | Tatárszentgyörgy | Summer |
| REF181 | 25 | JN859401 | *Ephedra distachya* | Fülöpháza | Fall |
| REF182 | 25 | JN859402 | *Juniperus communis* | Tatárszentgyörgy | Fall |
| REF183 | 25 | JN859403 | *Juniperus communis* | Bugac | Fall |
| REF184 | 25 | JN859404 | *Juniperus communis* | Tatárszentgyörgy | Fall |
| REF185 | 25 | JN859405 | *Juniperus communis* | Bugac | Fall |
| REF186 | 25 | JN859406 | *Juniperus communis* | Bugac | Fall |
| REF187 | 25 | JN859407 | *Juniperus communis* | Bugac | Fall |
| REF188 | 25 | JN859408 | *Ailanthus altissima* | Tatárszentgyörgy | Fall |
| REF189 | 25 | JN859409 | *Juniperus communis* | Fülöpháza | Fall |
| REF190 | 25 | JN859410 | *Ailanthus altissima* | Tatárszentgyörgy | Fall |
| REF191 | 25 | JN859411 | *Juniperus communis* | Bugac | Summer |
| REF192 | 25 | JN859412 | *Juniperus communis* | Bugac | Summer |
| REF193 | 25 | JN859413 | *Juniperus communis* | Bugac | Summer |
| REF194 | 25 | JN859414 | *Juniperus communis* | Bugac | Summer |
| REF195 | 25 | JN859415 | *Asclepias syriaca* | Fülöpháza | Summer |
| REF196 | 25 | JN859416 | *Asclepias syriaca* | Fülöpháza | Summer |
| REF197 | 25 | JN859417 | *Juniperus communis* | Bugac | Spring |
| REF198 | 25 | JN859418 | *Juniperus communis* | Tatárszentgyörgy | Spring |
| REF199 | 25 | JN859419 | *Juniperus communis* | Fülöpháza | Spring |
| REF200 | 25 | JN859420 | *Juniperus communis* | Bugac | Spring |
| REF201 | 25 | JN859421 | *Juniperus communis* | Tatárszentgyörgy | Spring |
| REF202 | 25 | JN859422 | *Juniperus communis* | Bugac | Spring |
| REF203 | 26 | JN859423 | *Populus alba* | Fülöpháza | Summer |
| REF204 | 26 | JN859424 | *Ailanthus altissima* | Tatárszentgyörgy | Summer |
| REF205 | 26 | JN859425 | *Juniperus communis* | Tatárszentgyörgy | Fall |
| REF206 | 26 | JN859426 | *Ailanthus altissima* | Fülöpháza | Summer |
| REF207 | 26 | JN859427 | *Ailanthus altissima* | Fülöpháza | Summer |
| REF208 | 26 | JN859428 | *Medicago minima* | Fülöpháza | Summer |
| REF209 | DSE-14 | JN859429 | *Ailanthus altissima* | Tatárszentgyörgy | Fall |
| REF210 | DSE-14 | JN859430 | *Ailanthus altissima* | Tatárszentgyörgy | Fall |
| REF211 | DSE-14 | JN859431 | *Ailanthus altissima* | Fülöpháza | Summer |
| REF212 | 27 | JN859432 | *Populus alba* | Fülöpháza | Summer |
| REF213 | 27 | JN859433 | *Ephedra distachya* | Fülöpháza | Fall |
| REF214 | 27 | JN859434 | *Ailanthus altissima* | Tatárszentgyörgy | Fall |
| REF215 | 27 | JN859435 | *Medicago minima* | Fülöpháza | Summer |
| REF216 | 27 | JN859436 | *Medicago minima* | Fülöpháza | Summer |
| REF217 | 27 | JN859437 | *Medicago minima* | Fülöpháza | Summer |
| REF218 | 27 | JN859438 | *Medicago minima* | Fülöpháza | Summer |

| | | | | | |
|---|---|---|---|---|---|
| REF219 | 27 | JN859439 | *Juniperus communis* | Tatárszentgyörgy | Summer |
| REF220 | 27 | JN859440 | *Juniperus communis* | Tatárszentgyörgy | Spring |
| REF221 | 27 | JN859441 | *Helianthemum ovatum* | Fülöpháza | Summer |
| REF222 | 27 | JN859442 | *Ailanthus altissima* | Fülöpháza | Summer |
| REF223 | 27 | JN859443 | *Ephedra distachya* | Fülöpháza | Fall |
| REF224 | 27 | JN859444 | *Ailanthus altissima* | Fülöpháza | Summer |
| REF225 | 27 | JN859445 | *Asclepias syriaca* | Fülöpháza | Summer |
| REF226 | 27 | JN859446 | *Ailanthus altissima* | Fülöpháza | Summer |
| REF227 | 27 | JN859447 | *Ailanthus altissima* | Fülöpháza | Summer |
| REF228 | 27 | JN859448 | *Ailanthus altissima* | Fülöpháza | Summer |
| REF229 | 27 | JN859449 | *Juniperus communis* | Tatárszentgyörgy | Summer |
| REF230 | 27 | JN859450 | *Juniperus communis* | Bugac | Summer |
| REF231 | 27 | JN859451 | *Juniperus communis* | Bugac | Summer |
| REF232 | 27 | JN859452 | *Juniperus communis* | Bugac | Fall |
| REF233 | 27 | JN859453 | *Juniperus communis* | Bugac | Fall |
| REF234 | 27 | JN859454 | *Juniperus communis* | Bugac | Fall |
| REF235 | 27 | JN859455 | *Helianthemum ovatum* | Fülöpháza | Summer |
| REF236 | 27 | JN859456 | *Populus alba* | Fülöpháza | Summer |
| REF237 | 27 | JN859457 | *Juniperus communis* | Fülöpháza | Summer |
| REF238 | 27 | JN859458 | *Juniperus communis* | Tatárszentgyörgy | Summer |
| REF239 | 27 | JN859459 | *Ailanthus altissima* | Tatárszentgyörgy | Fall |
| REF240 | 27 | JN859460 | *Medicago minima* | Fülöpháza | Summer |
| REF241 | 27 | JN859461 | *Juniperus communis* | Bugac | Summer |
