# Supplementary material for: The Dark Side Is Not Fastidious – Dark Septate Endophytic Fungi of Native and Invasive Plants of Semiarid Sandy Areas
Source: PLoS One. 2012 Feb 29;7(2):e32570. doi: 10.1371/journal.pone.0032570 (PMC3290574; doi:10.1371/journal.pone.0032570)
Supplement: Table S2 — Closest BLAST matches of representative isolates of DSE groups. Name, source, provenance country, query coverage and max ident of sequences from GenBank are indicated. Abbreviations: uncultured (u.), clone (c.), strain (st.), Host (H), Isolation Source (IS), Tissue Type (TT), Environmental Sample (ES). (PDF) [file pone.0032570.s007.pdf]

Table S2 Closest BLAST matches of representative isolates of DSE groups

DSE group	Isolate	Closest sequence matches	Source / Host	Country	Coverage	Similarity
**1**	**REF025**	u. mycorrhizal ascomycete (AY634148)	H: *Epipactis atrorubens*, TT: mycorrhizal root section, ES	Germany	100	99
		Cadophora sp. (FJ666349)	H: *Populus tremuloides*	Canada	99	98
		Cadophora sp. (GU212389)	IS: box packing material sample	Antarctica	100	97
		Cadophora sp. (DQ317329)	IS: Ross Sea region of Antarctica	Antarctica	100	97
		Cadophora luteo-olivacea is. (FJ486274)			100	97
		u. *Cadophora* c. (EU517022)	IS: soil, ES	Austria	97	98
		Cadophora luteo-olivacea st. (DQ404348)	IS: *Actinidia deliciosa* discolored wood	Italy	100	96
		Cadophora luteo-olivacea st. (GU128589)	IS: apple fruit	Italy	100	96
		Cadophora luteo-olivacea is. (GQ214536)			100	96
		Cadophora luteo-olivacea st. (FJ430742)	IS: soil	Czech Rep.	100	96
		u. ascomycete is. (AY833035)	H: *Cephalanthera damasonium*, IS: orchid mycorrhiza, ES	France	96	97
	**REF039**	u. soil fungus c. (EU490132)	IS: savanna soil (0 to 10 cm) under *Prosopis glandulosa*, ES	USA: TX	98	97
		Cadophora luteo-olivacea (HM116748)	H: *Actinidia deliciosa* cv. Hayward	New Zealand	98	96
		Cadophora luteo-olivacea st. (DQ404349)			97	97
		Rhexocercosporidium sp. (DQ275614)	H: *Panax ginseng*	Canada	97	96
		u. soil fungus c. (DQ420925)	IS: soil, ES	USA: MN	97	96
		u. *Leptodontidium* is. (FJ378720)	H: *Kobresia* sp., ES	China	97	96
		dark septate endophyte (AF168783)	H: *Ranunculus adoneus*	USA: CO	99	95
		u. ascomycete is. (DQ182423)	IS: host roots, H: *Cephalanthera longifolia*, ES		97	96
		Leptodontidium orchidicola st. (AF486133)	H: *Platanthera hyperborea*		97	96
**2**	**REF058**	u. fungus c. (GQ921746)	IS: soil, ES	Australia	100	99
		u. Helotiales c. (FJ197867)	IS: primary successional glacier foreland soil, ES	Austria	95	99
		u. fungus c. (EU292653)	IS: soil, ES	USA: AK	100	97
		u. Helotiales c. (GU327458)	H: *Epipactis helleborine*, IS: mycorrhizal seedling, ES	Czech Rep.	92	99
		u. soil fungus (GU083256)	IS: total community (raw soil DNA extraction), ES	USA: AK	100	97
		u. fungus c. (FJ553784)	IS: forest soil, ES	Canada	100	96
		u. Helotiales is. (DQ182427)	H: *Cephalanthera longifolia*, IS: host roots, ES		100	96
		Ericoid mycorrhizal sp. (AY046400)	IS: *Quercus ilex* areas	Italy	99	96
		u. Ascomycota c. (FJ440888)	IS: roots of *Pyrola* plants, ES	USA: OR/CA	100	96
		u. ectomycorrhiza (Helotiales) is. (EF644169)	IS: ectomycorrhizal root tips of *Populus tremula*, TT: ectomycorrhizal mantle, ES	Austria	97	96
		u. ectomycorrhiza (*Leohumicola verrucosa*) c. (DQ497978)	H: *Tsuga heterophylla*, IS: root, ES	Canada	100	94
		Leohumicola minima st. (AY706329)	IS: volcanic ash soil	Chile	98	94
		Fungal endophyte sp. (EU686206)	H: *Scapania* sp.	Wales	100	94
**3**	**REF078**	Fungal endophyte (AF373055)	H: *Rosmarinus officinalis*, IS: endomycorrhizae of host	Italy	100	99
		Fungal endophyte (AF373052)	H: *Pinus halepensis*, IS: from ectomycorrhizae of host	Italy	100	99
		u. endophytic fungus (AJ879648)	IS: root tips, TT: ectomycorrhiza, ES	Italy	100	99
		u. endophytic fungus c. (FJ524327)	IS: *Oryza granulate* root endophyte, ES	China	100	99
		Rhizopycnis vagum is. (HQ610506)	IS: diseased roots collected from farm fields, H: *Citrullus lanatus*	USA: IN	100	99
		Ascomyceta sp. (HM208718)	IS: hair root, H: *Rhododendron fortunei*	China	100	99
		Ascomycete sp. (AM944359)	IS: *Lycopersicum esculentum* disinfected roots	Colombia	100	99
		Fungal endophyte sp. (DQ459005)	H: *Dioscorea zingiberensis* rhizomes	China	100	99
		Fungal sp. (HM208743)	IS: hair root, H: *Rhododendron fortunei*	China	100	98
		Rhizopycnis vagum (AF022786) Holotype culture (TX951120)	IS: *Cucumis melo* var. *cantalupensis*	USA: TX	100	98
		Rhizopycnis sp. (DQ682600)	H: *Coffea arabica* roots		97	99
		Fungal endophyte sp. (EU250374)	IS: fruit of host, H: *Camptotheca acuminata*		100	95
		Pleosporales sp. (EU002891)	IS: peduncle endophyte, H: *Coffea arabica*, ES	USA: HI	100	95
		Fungal endophyte sp. (EU625406)	IS: mulberry roots		96	98
		Rhizopycnis sp. (FJ827623)	IS: *Coffea arabica* endophyte	Ethiopia	93	99
		Ascomycete sp. (EF672295)	H: *Coffea arabica* roots	USA	92	99
		Pleosporales sp. (FJ624263)	H: mangrove		95	94
		Rhizopycnis vagum st. (FJ582640)			100	93
**4**	**REF101**	*Pithomyces valparadisiacus* (EU552152)	H: *Protea lepidocarpodendron*	South Africa	60	85
		u. fungus c. (GQ921741)	IS: soil, ES	Australia	42	100
		u. fungus is. (GU564990)	H: *Populus tremula*, ES	Sweden	32	88
		Coniothyrium sp. (GU062312)	H: *Alnus incana*, IS: wood	Latvia	43	100
		u. fungus (FN397425)	IS: *T. melanosporum* truffle-ground soil, outside burnt area, ES	France	43	100
		u. Ascomycota c. (FJ553168)	IS: forest soil, ES	Canada	35	100
		Coniothyrium fuckelii is. (FJ228185)	H: *Fraxinus excelsior*	Sweden	43	100
		Ascomycete sp. (AJ972833)	IS: Marble Monument	Turkey	39	84
		Fungal endophyte (FN394698)	H: *Holcus lanatus*	Spain	27	93
		u. ascomycete c. (EU490122)	IS: savanna soil (0 to 10 cm) under *Prosopis glandulosa*, ES	USA	42	100
		Saccharicola sp. (GU973718)	IS: root-endophyte, H: sugarcane cultivar IMI-1	Brazil	42	100
		Coniothyrium fuckelii (AY904055)	H: *Rosa* sp., TT: stem	Mexico	42	100
		u. fungus (AM260897)	IS: peat, ES	UK	43	100
**5**	**REF104**	*Alternaria helianthi* st. (DQ156343)		India	100	90
		Alternaria helianthi (AY154713)	H: *Helianthus annus* (leaves)	Iran	100	90
		Alternaria leucanthemi st. (AF314586)			100	90
		Alternaria leucanthemi st. (AY372684)		China	99	90
		Alternaria helianthi is. (HM449994)	IS: seeds, H: *Helianthus annuus* cv. Bhanu	India	90	91

		u. fungus c. (EU516993)	IS: soil, ES	Austria	88	89
		Leptosphaeria lindquistii is. (HM003206)	H: *Helianthus annuus*, TT: mycelium	China	87	89
		u. fungus c. (FJ237205)	IS: in-growth mesh bags in snow covered alpine soil, ES	Austria	93	88
		Leptosphaeria maculans (M96663)			91	87
		u. fungus c. (GU817175)	H: *Bistorta vivipara* individual 3.1, IS: root system	Norway	93	87
		Leptosphaeria sp. (DQ093683)	IS: *Pinus sylvestris* decayed roots, ES	Lithuania	96	86
**6**	**REF106**	u. ascomycete c. (EF154351)	H: *Fagopyrum esculentum* roots, ES		100	100
		u. Ascomycota c. (HM162252)	IS: grass roots, ES	USA: TX	100	99
		u. ascomycete c. (EU358787)	H: *Capsicum annuum*, ES	Mexico	100	99
		u. ascomycete c. (EU177123)	H: *Capsicum annuum*, IS: roots	Mexico	100	99
		u. soil fungus c. (EU490117)	IS: savanna soil (0 to 10 cm) under *Prosopis glandulosa*, ES	USA TX	100	99
		Alternaria sp. (HQ630996)	H: *Miscanthus giganteus*	USA: IL	100	98
		Thielaviopsis basicola is. (GQ131878)	IS: peach rootstock, TT: mycelia	Italy	94	99
		Alternaria longissima is. (DQ865104)	H: *Linaria hegelmaieri*	Spain	95	99
	**REF109**	*Setophoma sacchari* (FN394730)	H: *Holcus lanatus*	Spain	100	100
		Hypocreales sp. (GQ923970)	IS: Ojuelos, H: *Bouteloua gracilis*	Mexico	100	99
		u. endophytic fungus c. (EF505610)	H: *Zea mays*, IS: leaves, stems, kernels, ES	USA	100	99
		Ascomycete sp. (EF672299)	H: *Coffea arabica*	Colombia	99	99
		Setophoma sacchari (AB499793)	H: *Allium fistulosum*	Japan	98	99
		Fungal endophyte st. (FJ450016)			100	99
**7**	**REF123**	u. soil fungus c. (EU480270)	IS: biological soil crust, ES	USA: NM	100	97
		u. root-associated fungus c. (EU144602)	IS: roots, H: *Bouteloua gracilis* , ES	USA: NM	100	97
		Root-associated fungal sp. (EU144366)	IS: roots, H: *Bouteloua gracilis* , ES	USA: NM	100	96
		u. root-associated fungus c. (FJ449545)	IS: roots, H: *Bouteloua gracilis*, ES	USA: NM	96	96
	**REF128**	u. soil fungus c. (EU480184)	IS: biological soil crust, ES	USA: NM	99	97
		u. root-associated fungus c. (EU144953)	IS: roots, H: *Bouteloua gracilis* , ES	USA: NM	99	97
		Pleosporales sp. (GQ923954)	IS: grasslands, H: *Bouteloua gracilis*	Canada: SK	100	97
		u. Pleosporales c. (GQ924050)	IS: roots from wind cave, H: *Bouteloua gracilis*	USA: SD	99	97
		Fungal sp. (FJ449549)	IS: roots, H: *Bouteloua gracilis*, ES	USA: NM	93	97
	**REF130**	u. root-associated fungus c. (EU145013)	IS: roots, H: *Bouteloua gracilis* , ES	USA: NM	100	97
		u. root-associated fungus c. (FJ361958)	IS: roots, H: *Bouteloua gracilis*, ES	USA: NM	100	97
		Root-associated fungal sp. (EU144367)	IS: roots, H: *Bouteloua gracilis* , ES	USA: NM	100	97
		u. soil fungus c. (EU479732)	IS: *Bouteloua gracilis* rhizosphere soil, ES	USA: NM	100	97
	**REF132**	u. root-associated fungus c. (FJ361994)	IS: roots, H: *Sporobolus cryptandrus*, ES	USA: NM	100	99
		u. soil fungus c. (EU480264)	IS: biological soil crust, ES	USA: NM	100	99
		u. root-associated fungus c. (EU145010)	IS: roots, H: *Bouteloua gracilis* , ES	USA: NM	100	99
		u. Pleosporales c. (GQ924025)	IS: roots from Konza, H: *Bouteloua gracilis*	USA: KS	100	99
		Pleosporales sp. (GQ923972)	IS: Oujelos, H: *Bouteloua gracilis*	Mexico	100	96
	**REF140**	u. root-associated fungus c. (EU144759)	IS: roots, H: *Bouteloua gracilis* , ES	USA: NM	98	99
		u. mycorrhizal fungus c. (AY929107)	IS: root; semi-arid grassland, H: *Stipa hymenoides*, ES	USA: UT	99	98
		u. Pleosporales c. (HQ389473)	IS: roots, H: *Sporobolus cryptandrus*, ES	USA: NM	96	99
		u. Pleosporales c. (GQ924053)	IS: roots from SNWR, H: *Bouteloua gracilis*	USA: NM	96	99
**8**	**REF144**	*Periconia macrospinosa* st. (FJ536208)	IS: roots of C_4 grasses	USA: KS	100	99
		u. Ascomycota c. (HM162309)	IS: grass roots, ES	USA: TX	100	99
		u. *Periconia* c. (GU055658)	IS: grassland soil	Austria	99	89
		Periconia macrospinosa (AJ246159)	IS: *Avena sativa* cv. Gerald roots	UK	95	100
		Pleosporales sp. (GQ923975)	IS: Ojuelos, H: *Bouteloua gracilis*	Mexico	95	99
		u. soil fungus c. (DQ420990)	IS: soil, ES	USA: MN	100	97
		Periconia sp. (HQ631028)	H: *Saccharum officinarum*	USA: LA	100	97
		Periconia macrospinosa (FN393421)	H: *Holcus lanatus* roots	Spain	92	99
		u. fungus c. (HQ260291)	IS: roots, H: Ericaceae, ES	USA: AK	100	97
		Massarina sp. (GQ141701)	IS: brown rice, H: rice		93	98
		Periconia sp. (HQ130695)	H: *Warburgia ugandensis*		96	97
		Massarina igniaria st. (EU715627)	IS: branch, H: *Taxus globosa*	Mexico	93	97
		u. fungus c. (GQ999275)	IS: air filter sample, ES		100	94
		Periconia macrospinosa st. (HQ328037)			93	97
		Periconia sp. (FJ903341)	IS: decayed wood, H: *Picea abies*	Latvia	90	97
		Fungal endophyte sp. (EU685980)	H: *Cheilolejeunea* sp.	Peru	93	96
		Massarina igniaria st. (GQ377480)	H: *Taxus globosa*, IS: branch	Mexico	91	97
		Periconia macrospinosa (AM262349)	H: *Dactylis glomerata*	Spain	81	100
		Fungal endophyte sp. (EU977233)	H: Macairea thyrsiflora	Peru	88	97
**9**	**REF148**	u. soil fungus c. (EU480177)	IS: biological soil crust, ES	USA: NM	100	100
		Embellisia sp. (AY345356)	H: *Hennediella heimii*	Antarctica	100	99
		u. root-associated fungus c. (FJ362231)	IS: roots, H: *Sporobolus cryptandrus*, ES	USA: NM	100	99
		Embellisia astragali st. (FJ914716)			100	99
		Embellisia sp. (AY864335)	H: *Hennediella heimii*	Antarctica	100	99
		Alternaria japonica (AY154703)	H: *Raphanus sativus* (leaves)	Iran	100	95
	**REF150**	*Embellisia* sp. (AF212309)		Antarctica	100	99
		u. soil fungus c. (EU480175)	IS: biological soil crust, ES	USA: NM	100	99
		u. root-associated fungus c. (EU144461)	IS: roots, H: *Bouteloua gracilis* , ES	USA: NM	96	99
		Embellisia tellustre st. (FJ357316)	H: *Oxytropis* spp.	USA: NM	97	99
		Embellisia allii (AY278840)			96	99
		Embellisia allii (AB477430)	H: *Allium sativum*	China	92	98
		Pleospora papaveracea is. (DQ885386)	H: *Papaver somniferum*	Spain	100	94
		Chalastospora gossypii (FN868458)	H: *Pinus halepensis*	Spain	100	92
**10**	**REF154**	u. fungus (FN397304)	IS: *Tuber melanosporum* truffle-ground soil, burnt area, ES	France	100	100

		Curvularia inaequalis (FM163616)	IS: nasal secretion	Italy	100	100
		u. Ascomycota c. (HM161938)	IS: grass roots, ES	USA: TX	100	99
		u. endophytic fungus c. (EF505555)	H: *Zea mays*, IS: leaves, stems, kernels, ES	USA	99	100
		Curvularia coicicola (AB453880)	IS: *Coicis lacryma-jobi* L.	China	100	99
		Curvularia inaequalis st. (HM101095)			98	100
		Curvularia inaequalis st. (AF120261)			98	99
		u. ascomycete c. (EU489922)	IS: savanna soil (0 to 10 cm) under C4 midgrasses, ES	USA	100	97
		Curvularia inaequalis (AF313409)			100	97
		u. soil fungus c. (DQ420883)	IS: soil, ES	USA: MN	100	97
		Curvularia geniculata (AB245085)	H: *Agrostis stronifela*	Japan	99	97
		Curvularia inaequalis st. (AY941256)			92	100
**11**	**REF158**	*Microdochium bolleyi* (AM502264)			100	100
		Microdochium bolleyi st. (GU566298)	IS: rhizosphere, H: *Phalaris arundinacea*	Czech Rep.	100	100
		Microdochium sp. (AJ279489)	H: *Phragmites australis* roots	Germany	100	100
		Microdochium sp. (HQ630981)	H: *Miscanthus giganteus*	USA: IL	100	99
		u. fungus (FN391313)	IS: *Tuber melanosporum* truffle-ground soil, burnt area, ES	France	100	99
		Xylariales sp. (EU755004)	IS: soil and roots of oilseed rape, ES		100	99
		u. endophytic fungus c. (EF505625)	H: *Zea mays*, IS: leaves, stems, kernels, ES	USA	100	99
		Microdochium sp. (FJ439588)	IS: soil artificially contaminated with pyrene	UK	98	100
		Ascomycete sp. (AY243053)	H: *Ammophila arenaria*, IS: plants grown in greenhouse in sterilized and unsterilized soil	USA: CA	96	99
		Microdochium bolleyi (AM924150)	H:*Elymus farctus*	Spain	92	100
		u. Xylariales c. (GQ924061)	H: *Bouteloua gracilis*, IS: roots from wind cave, ES	USA: SD	92	99
		Microdochium bolleyi is. (HM007082)	IS: *Stipa grandis*, IS: roots	China	90	99
		Microdochium sp. (FJ536210)	H: roots of C_4 grasses	USA: KS	92	100
		u. Xylariales c. (DQ317371)	IS: Ross Sea region of Antarctica, ES	Antarctica	66	100
**12**	**REF165**	Fungal sp. (HM036626)	H: *Pinus sylvestris*, IS: root tip	Lithuania	83	99
		Thermoascus crustaceus st. (EU021597)			99	87
		Byssochlamys verrucosa (DQ073329)		Australia	99	87
		Thermoascus crustaceus (U18353)	TT: mycelium, dev stage: hyphal		99	87
		Penicillium inflatum (AJ608959)	IS: plasticised polyvinylchloride	Bulgaria	99	87
		Penicillium inflatum st. (AF033393)			99	87
		Thermoascus crustaceus st. (FJ389925)	H: *Parthenium argentatum*	USA	98	87
		Aspergillus wenti is. (FJ537089)			95	88
		Aspergillus wentii is. (EF652158)			95	88
		Aspergillus dimorphicus (FR727119)	IS: phylloplane	Czech Rep.	95	88
		Aspergillus heteromorphus (AJ876879)	IS: culture contaminant	Brazil	99	87
		Talaromyces leycettanus st. (AF454080)			99	87
**13**	**REF168**	u. *Plectosphaerella* c. (GU327453)	H: *Epipactis helleborine*, IS: mycorrhizal seedling, ES	Czech Rep.	100	99
		Phyllachoraceae sp. (EU755008)	IS: soil and roots of oilseed rape, ES		100	99
		u. fungus (FM875855)	IS: biofilter reactor, ES		100	99
		Plectosphaerella sp. (FJ430715)		Czech Rep.	100	99
		u. ascomycete c. (AY615882)	IS: field rhizosphere, ES	Nedherlands	100	99
		u. ascomycete (AM901905)	IS: house dust, ES	Finland	98	99
		u. fungus c. (GQ921802)	IS: soil, ES	Australia	98	99
		Plectosphaerella cucumerina st. (EF495236)	IS: *Paris polyphylla* var. *yunnanensis* rhizomes	China	100	99
		Plectosphaerella sp. (GQ407099)	H: *Musa acuminata*, IS: root, N: fungal endophyte	Malaysia	100	98
		Plectosphaerella cucumerina st. (DQ779781)	IS: soil		98	99
		Plectosphaerella cucumerina (AJ492873)	H: potato cyst nematode	UK	100	98
		Plectosphaerella cucumerina (FM178318)		Australia	96	99
		Plectosphaerella sp. (HQ008920)	H: *Arrabidaea candicans*	Ecuador	100	98
		Plectosphaerella cucumerina (AB469880)	H: *Homo sapiens* cornea		100	98
		Colletotrichum pisi st. (EU400150)			100	97
**14**	**REF210**	*Fusarium* sp. (HQ166538)	H: *Buxus sempervirens*, IS: infected leaves	Switzerland	100	100
		Hypocreales sp. (GQ923966)	H: *Bouteloua gracilis*, IS: wind cave	USA: SD	100	100
		Fusarium acuminatum st. (HM068325)			100	100
		Fusarium sp. (GU480954)	H: wheat, IS: seeds	Mexico	100	100
		Fusarium sp. (GQ505464)		USA: TX	100	100
		Fungal sp. (GQ866861)	H: *Spiranthes sinensis* (Pers.) Ames	China	100	100
		u. root-associated fungus c. (FJ362249)	H: *Yucca glauca*, IS: roots, ES	USA: NM	100	100
		Fusarium solani (AB470848)	H: *Larix gmelinii* var. principis-rupprechtii	China	100	100
		Fusarium sp. (EU910079)	H: *Cicer arietinum*		100	100
		Fusarium tricinctum (FJ233196)	H: kiwifruit	China	100	100
		Hypocreales sp. (EU754934)	IS: soil and roots of oilseed rape, ES		100	100
		Gibberella avenacea is. (EU255801)			100	100
		u. *Fusarium* c. (EU003042)	H: apple seedling, IS: roots, ES		100	100
		u. soil fungus c. (DQ421028)	IS: soil, ES	USA: MN	100	100
		Fusarium tricinctum (AY188923)			100	100
		Fungal endophyte is. (EU167917)	H: *Picrorhiza kurrooa*		99	100
		u. *Gibberella* (FM178250)	IS: malthouse waste water, ES		100	99
		Gibberella pulicaris st. (FJ481029)	H: poplar, IS: root		100	99

Name, source, provenance country, query coverage and max ident of sequences from GenBank are indicated. Abbreviations: uncultured (u.), clone (c.), strain (st.), Host (H), Isolation Source (IS), Tissue Type (TT), Environmental Sample (ES)
